# Acyl-CoA synthetase 6 is required for brain docosahexaenoic acid retention and neuroprotection during aging

**DOI:** 10.1172/jci.insight.144351

**Published:** 2021-06-08

**Authors:** Regina F. Fernandez, Andrea S. Pereyra, Victoria Diaz, Emily S. Wilson, Karen A. Litwa, Jonatan Martínez-Gardeazabal, Shelley N. Jackson, J. Thomas Brenna, Brian P. Hermann, Jeffrey B. Eells, Jessica M. Ellis

**Affiliations:** 1 Department of Physiology, Brody School of Medicine, and East Carolina Diabetes and Obesity Institute, East Carolina University, Greenville, North Carolina, USA.; 2 Department of Biology, University of Texas San Antonio, San Antonio, Texas, USA.; 3 Department of Anatomy and Cell Biology, Brody School of Medicine, East Carolina University, Greenville, North Carolina, USA.; 4 Department of Pharmacology, University of Basque Country, Leioa, Spain.; 5 Structural Biology Core, Intramural Research Program, National Institute on Drug Abuse, NIH, Baltimore, Maryland, USA.; 6 Departments of Pediatrics, Chemistry, and Nutrition and; 7 Dell Pediatric Research Institute, Dell Medical School, University of Texas at Austin, Austin, Texas, USA.

**Keywords:** Inflammation, Metabolism, Behavior, Eicosanoids, Neurological disorders

## Abstract

The omega-3 fatty acid docosahexaenoic acid (DHA) inversely relates to neurological impairments with aging; however, limited nondietary models manipulating brain DHA have hindered a direct linkage. We discovered that loss of long-chain acyl-CoA synthetase 6 in mice (*Acsl6^–/–^*) depletes brain membrane phospholipid DHA levels, independent of diet. Here, *Acsl6^–/–^* brains contained lower DHA compared with controls across the life span. The loss of DHA- and increased arachidonate-enriched phospholipids were visualized by MALDI imaging predominantly in neuron-rich regions where single-molecule RNA in situ hybridization localized *Acsl6* to neurons. ACSL6 is also astrocytic; however, we found that astrocyte-specific ACSL6 depletion did not alter membrane DHA because astrocytes express a non–DHA-preferring ACSL6 variant. Across the life span, *Acsl6^–/–^* mice exhibited hyperlocomotion, impairments in working spatial memory, and increased cholesterol biosynthesis genes. Aging caused *Acsl6^–/–^* brains to decrease the expression of membrane, bioenergetic, ribosomal, and synaptic genes and increase the expression of immune response genes. With age, the *Acsl6^–/–^* cerebellum became inflamed and gliotic. Together, our findings suggest that ACSL6 promotes membrane DHA enrichment in neurons, but not in astrocytes, and is important for neuronal DHA levels across the life span. The loss of ACSL6 impacts motor function, memory, and age-related neuroinflammation, reflecting the importance of neuronal ACSL6-mediated lipid metabolism across the life span.

## Graphical abstract

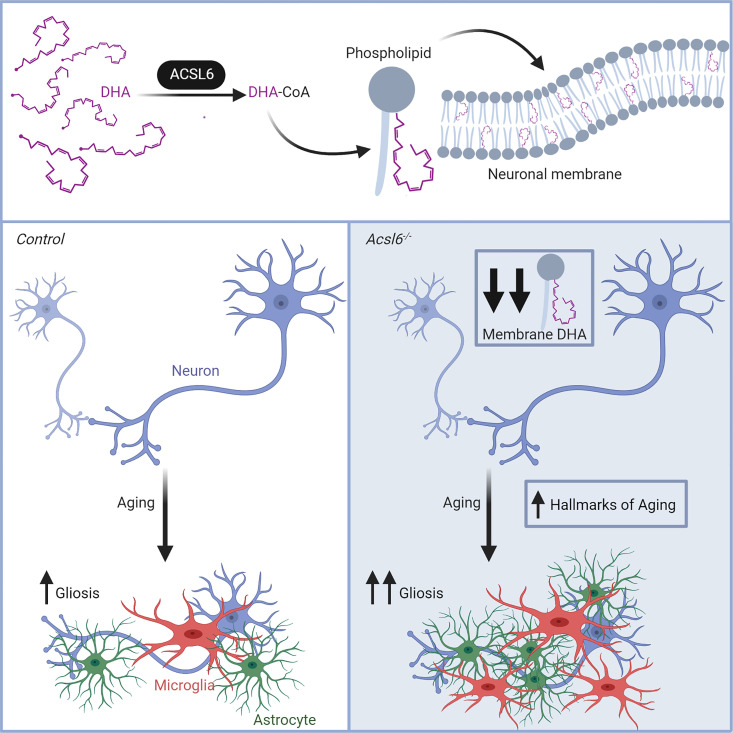

## Introduction

The brain is heavily enriched with phospholipids, making it the second most fatty organ of the body. Phospholipids are the major component of membranes and generally contain high levels of polyunsaturated fatty acids (PUFAs); thus, the brain is highly enriched with PUFAs, to a level 3- to 4-fold higher than other tissues (1, 2). Several PUFAs are essential, meaning they cannot be synthesized endogenously, but instead, must be obtained from the diet. Essential fatty acid–derived PUFAs enriched in the brain include the omega-6 arachidonic acid (AA, 20:4n6) and the omega-3 docosahexaenoic acid (DHA, 22:6n3) (1, 3). While omega-6 fatty acids are heavily enriched in Westernized diets, the omega-3 DHA is poorly represented (4, 5). Low dietary DHA intake is compounded by aging-related brain DHA decline (6, 7). Both low dietary DHA intake and low brain DHA levels are associated with the development of numerous age-related neurological diseases and disorders characterized by neuroinflammation and cognitive decline (8–12). Reciprocally, DHA intake through diet and/or supplementation confers protection against age-related neurological decline in part due to its ability to serve as a precursor to proresolving lipid mediators that ameliorate inflammation and DHA’s ability to regulate membrane biophysical properties (13, 14). These benefits of DHA serve as a barrier to the brain’s susceptibility to neuroinflammation and cognitive decline with age.

While it has been established that the brain contains high levels of DHA and that brain DHA is correlated with brain health (15, 16), the direct linkage to health and regulatory mechanisms that enable DHA incorporation into brain membrane lipids remains unclear. Recently, we discovered that long-chain acyl-CoA synthetase 6 (ACSL6) is a major regulator of DHA enrichment in the brain (17, 18). The critical role for ACSL6 in brain DHA enrichment was exemplified in *Acsl6*-deficient mice (*Acsl6^–/–^*) by significant reductions (35%–72%) in DHA-containing phospholipids (17). *Acsl6* is one of the 26-member family of acyl-CoA synthetase enzymes that ligate a CoA to a fatty acid, generating an acyl-CoA (19). This enzymatic activity both traps free fatty acids within cells and also activates fatty acids for subsequent intracellular metabolism. Each acyl-CoA synthetase enzyme has unique enzymatic preferences, regulatory mechanisms, and expression profiles across cell types, properties that contribute to cell type–specific lipid metabolism and composition (20). Here, we investigated how the loss of ACSL6 and the consequent DHA deficiency and AA enrichment within the brain affects aging. We found that ACSL6 deficiency impaired spatial memory and resulted in hyperlocomotion and age-related cerebellar neuroinflammation. Together, these data demonstrate that ACSL6-mediated DHA metabolism is required for age-related neuroprotection.

## Results

### ACSL6 is critical for retaining DHA during aging.

**Figure 1:**
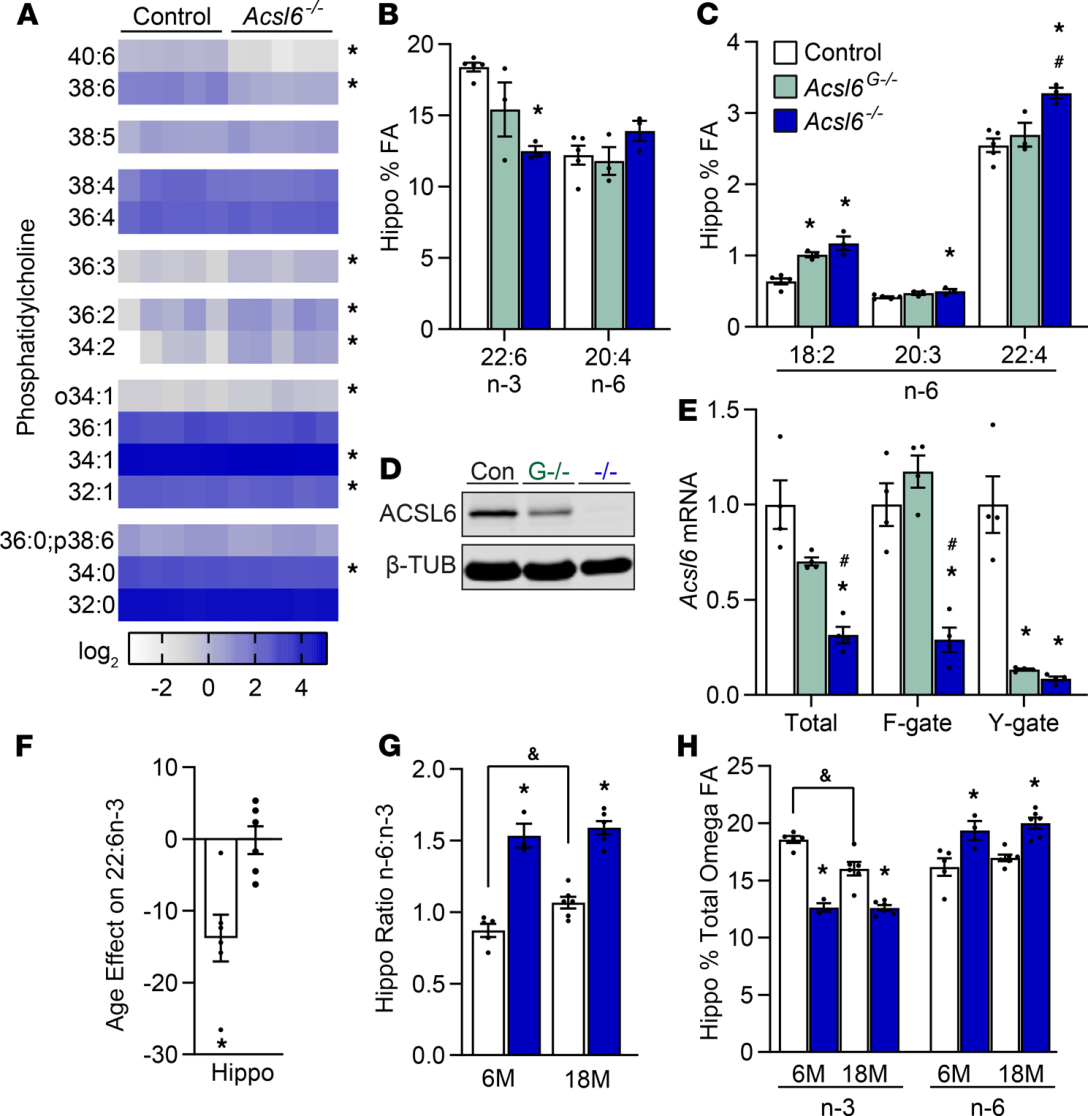
Effects of *Acsl6* loss on hippocampal DHA content during aging. (**A**) Heatmap of PCs clustered by degree of saturation in the control and *Acsl6*^–/–^ hippocampus, *n* = 5. Data are presented as percent of ion intensity distribution; species more than 0.5% of total are shown. (**B** and **C**) Percent of total hippocampal fatty acid content in 6-month-old control, *Acsl6^G–/–^*, and *Acsl6^–/–^* mice; *n* = 3–6. (**D**) Hippocampal immunoblot against ACSL6 and (**E**) cerebellar gate domain-targeted RT-PCR in control, *Acsl6*^G–/–^, and *Acsl6^–/–^* mice; *n* = 4. (**F**) Percent change of DHA from 6 to 18 months in the control and *Acsl6^–/–^* hippocampus; *n* = 3–6. (**G**) Omega-6 to omega-3 ratio. (**H**) Percent of omega-3 and omega-6 total fatty acids in 6- and 18-month-old control and *Acsl6^–/–^* hippocampus, *n* = 3–6. Data are shown as the mean ± SEM; *compared with control, ^&^by age within genotype, ^#^compared with *Acsl6^G–/–^*, *P* ≤ 0.05 (**B**, **C**, and **E**) by 1-way ANOVA with Tukey’s post hoc test, (**G** and **H**) by 2-way ANOVA with Sidak’s post hoc test, and (**F**) by 2-tailed Student’s *t* test.

Brain DHA content inversely correlates with the risk of age-related neurological diseases, disorders, and cognitive decline (6, 21, 22). We previously demonstrated that ACSL6 is responsible for enriching the brain and testes with DHA within membrane phospholipids (17, 23). Here, we measured brain lipids in the control and *Acsl6^–/–^* hippocampus, a region important for aging-sensitive functions such as memory and learning. Broad lipidomic profiling of phosphatidylcholine (PC) species demonstrated reductions in predicted DHA-enriched PCs (38:6 and 40:6) and increases in several PCs containing 1-, 2-, or 3-unsaturated bonds (Figure 1A). We next confirmed that the loss of highly unsaturated PCs was due to DHA deficiency by performing total fatty acid analysis to discover a 32% reduction in DHA (Figure 1B and Supplemental Table 1; supplemental material available online with this article; https://doi.org/10.1172/jci.insight.144351DS1). Other 22-carbon-long fatty acids were not reduced by the loss of ACSL6, and in fact, adrenic acid (22:4n6) levels were significantly increased (Figure 1C and Supplemental Table 1). Coincident with the reduction in relative DHA levels in *Acsl6^–/–^* were reciprocal increases in low-abundance fatty acids such as dihomo-γ-linolenic acid (20:3n6) and linoleic acid (18:2n6) and a statistically insignificant rise in the high-abundance fatty acid AA (20:4n6) (Figure 1, B and C, and Supplemental Table 1). These data suggest that ACSL6 exhibits a substrate specificity to DHA rather than a generalized capacity to metabolize 22-carbon-long fatty acids and confirms our previous findings that the loss of ACSL6 results in a DHA deficit.

A number of results in the literature indicate that astrocytes express ACSL6 (20, 24–28). To directly test the role of astrocytic ACSL6, we generated astrocyte-specific ACSL6-deficient mice using the glial fibrillary acidic protein (GFAP) promoter Cre driver (*Acsl6^G–/–^*) (17). Surprisingly, astrocyte-specific ACSL6 KOs exhibited minimal changes in fatty acid composition in the brain, most notably, unchanged DHA levels (Figure 1, B and C). However, total saturated fatty acids were significantly reduced, and linoleic acid (LA, 18:2n6) and eicosadienoic acid (20:2n6) were elevated (Figure 1C and Supplemental Table 1). A partial loss of ACSL6 protein in the *Acsl6^G–/–^* hippocampus was confirmed by Western blot and reiterates that a fraction of brain ACSL6 is expressed in astrocytes (Figure 1D). However, the lack of intermediary fatty acid profile phenotype between the complete and astrocytic KO models suggests differential metabolic roles for ACSL6 found in astrocytes compared with nonastrocytes. Therefore, the expression of ACSL6 variants, with differential fatty acid binding motifs, was assessed in the models (29). Targeted real-time PCR (RT-PCR) primers demonstrated that while total KO mice lost expression of both F- and Y-gate domains, the astrocyte-specific loss of ACSL6 retained expression of the F-gate (DHA-preferring) but lost the Y-gate (non–DHA-preferring) domain (Figure 1E). These data demonstrate that astrocytes do not express the DHA-preferring ACSL6 variant, thereby explaining the lack of DHA reduction in astrocyte-specific ACSL6-KO mice.

DHA levels naturally decline in the brain across the life span, a phenomenon predicted to increase the risk of age-related neurological impairments. However, the mechanistic underpinnings causing such age-related loss in brain DHA remain elusive. The fatty acid content of aged compared with young mice showed a decrease in DHA by 14% by aging (Figure 1F), consequently reducing total omega-3 fatty acids and increasing the omega-6/omega-3 ratio (Figure 1, G and H, and Supplemental Table 1). In *Acsl6^–/–^*, age did not affect the increased omega-6/omega-3 ratio (Figure 1G and Supplemental Table 1). Unlike in control mice, the *Acsl6^–/–^* hippocampus resisted aging-related reductions in brain DHA (Figure 1F). Together, these data suggest that nonastrocytic *Acsl6* is critical for enriching the brain with DHA throughout the life span.

### ACSL6 is expressed in mature neurons and drives neuronal membrane DHA enrichment.

**Figure 2:**
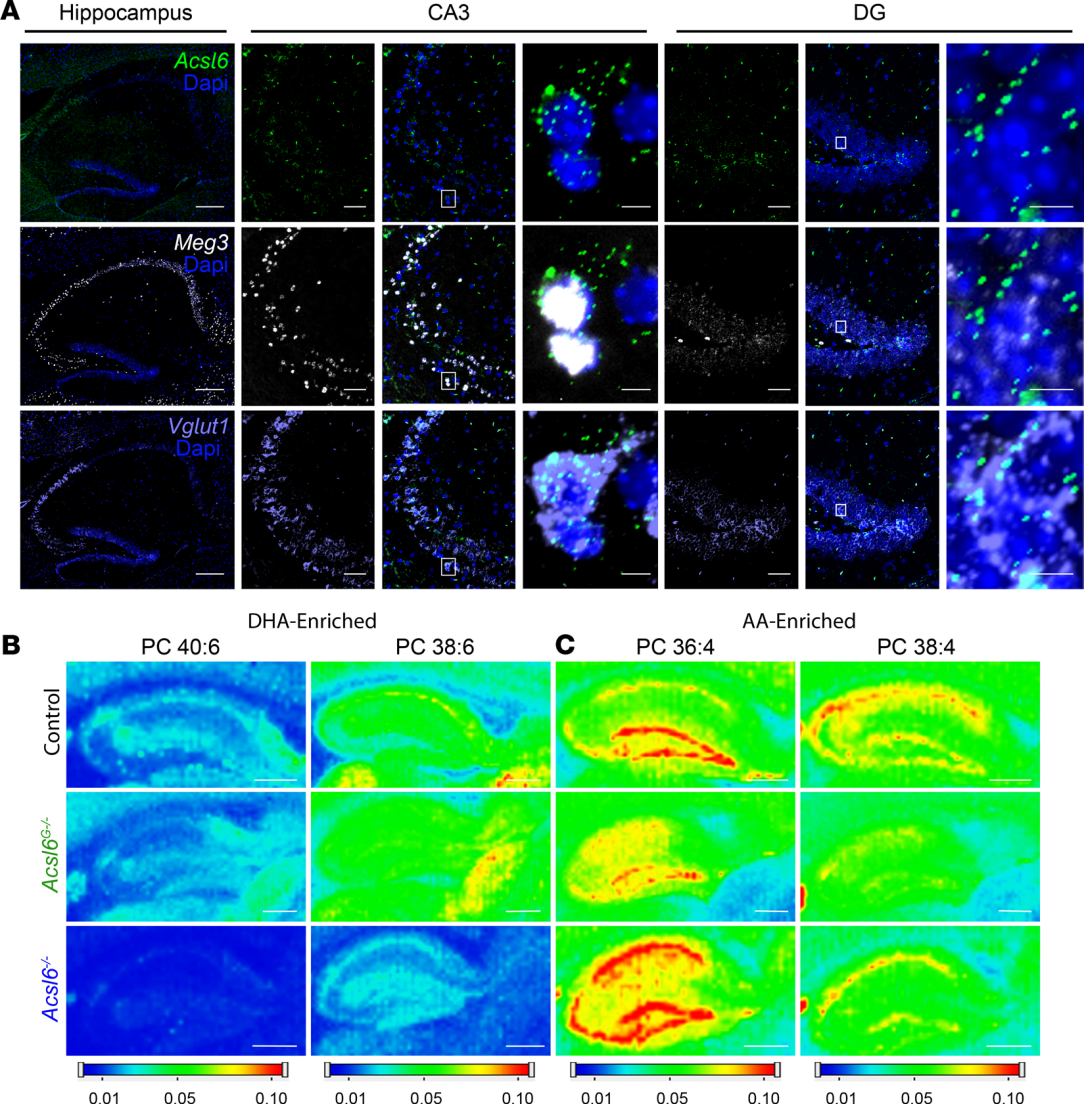
*Acsl6* is expressed in neurons and mediates brain DHA enrichment. (**A**) *Acsl6* detection by smFISH in the hippocampus and CA3 and DG regions; blue, nuclei; green, *Acsl6*; white, maternally expressed gene 3 (*Meg3*, pseudo pan-neuronal nuclear marker); and purple, *Vglut1*. White box indicates area of inset (scale bars: hippocampus, 200 μm; CA3 and DG, 50 μm; inset, 5 μm). Lipid imaging by MALDI of predicted (**B**) DHA-containing PCs (PC 40:6 *m/z* = 872.5566, [M + K] + and PC 38:6 *m/z* = 844.5253 [M + K]^+^) and (**C**) AA-containing PCs (PC 36:4 *m/z* = 820.5253 [M + K] + and PC 38:4 *m/z* = 848.5566 [M+K]^+^) in the control, *Acsl6^G–/–^*, and *Acsl6^–/–^* hippocampus (scale bars: 500 μm).

To better understand the spatial resolution and the cell type–specific contributions of *Acsl6* to lipid metabolism in the brain, we performed single-molecule RNA in situ hybridization (smFISH). *Acsl6* transcripts were evident across the hippocampus but particularly enriched in vesicular glutamate transporter 1–positive (Vglut1-positive) pyramidal neurons of the CA3 and dentate gyrus (Figure 2A). We next sought to determine the alignment between *Acsl6* expression and the spatial distribution of phospholipid acyl-chain composition using lipid mapping. Mapping of hippocampal phospholipids in control mice revealed DHA-enriched (40:6 and 38:6) and AA-enriched (36:4 and 38:4) PC particularly abundant in the neuron-rich CA1, CA2, CA3, and dentate gyrus regions (Figure 2, B and C). Total ACSL6 loss greatly reduced DHA-enriched phospholipids (PC40:6 and PC38:6) across the entire hippocampus (Figure 2B). Consequently, AA-enriched PC36:4 increased in the neuron-rich dentate gyrus, CA1, and CA3 regions (Figure 2C). Astrocyte-specific ACSL6 deletion showed a negligible impact on DHA-enriched phospholipids’ lipid images (Figure 2B). These data suggest that nonastrocytic ACSL6 enriches the hippocampus with membrane DHA, particularly in neuron-rich regions where ACSL6 is highly abundant.

### ACSL6 loss impairs short-term spatial working memory and induces hyperlocomotion.

**Figure 3:**
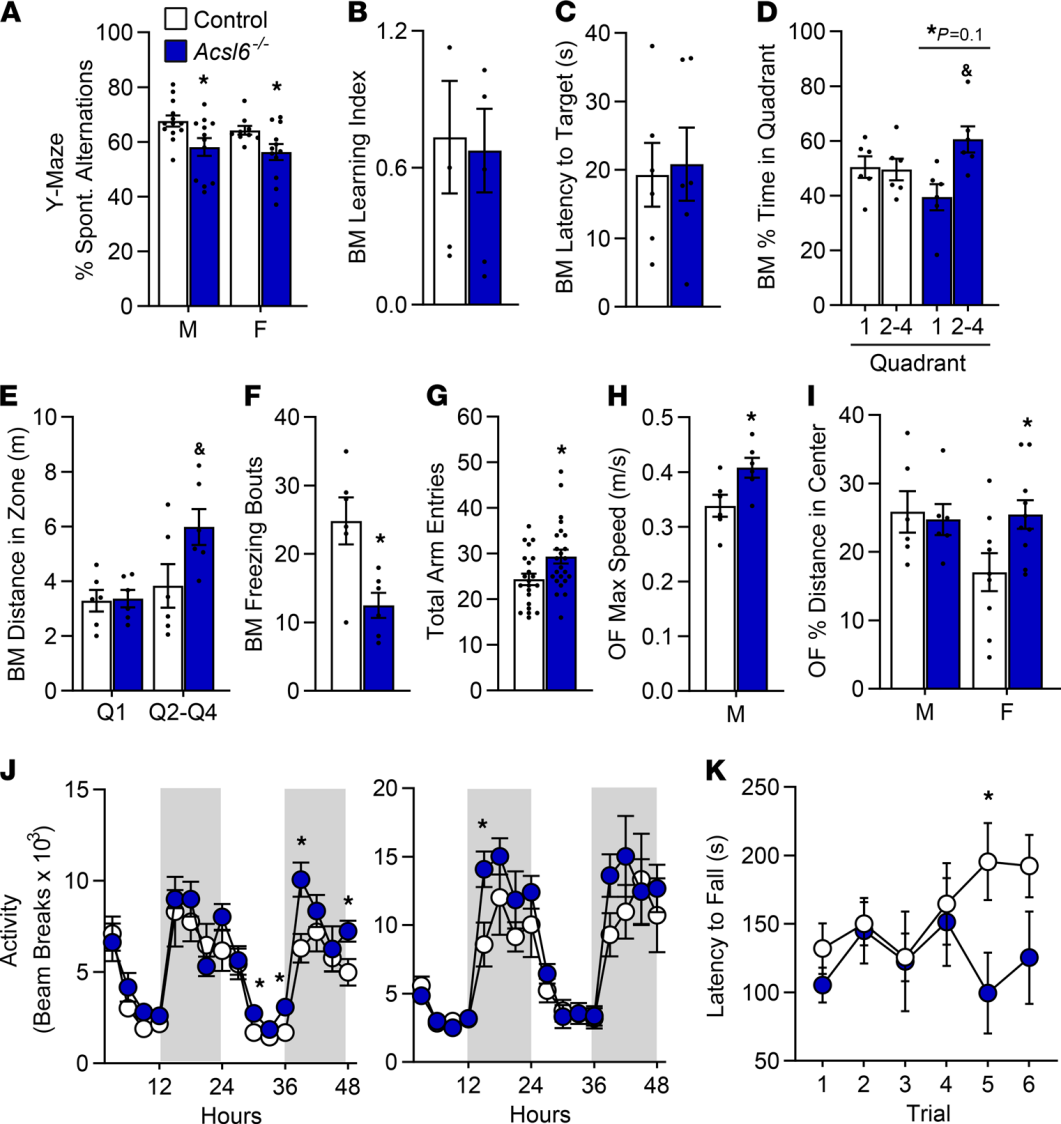
*Acsl6* exhibits reduced short-term spatial working memory and induces hyperlocomotion. (**A**) Y-maze percent of spontaneous alternations of 2-month-old female (F) and male (M) control and *Acsl6^–/–^* mice; *n* = 22–24. Hippocampus-dependent spatial memory and learning were assessed using the BM: (**B**) learning index during training, (**C**) latency to first enter target zone, (**D**) percent of time, (**E**) distance spent in each quadrant (Q) 1 or Q2–Q4, and (**F**) freezing bouts during the probe trial for 18-month-old control and *Acsl6^–/–^* males; *n* = 6. (**G**) Total entries in the Y-maze for 2-month-old control and *Acsl6^–/–^* males and females; *n* = 22–24. (**H**) Maximum speed and (**I**) percent of distance traveled in the center of the open field (OF) achieved over a 10-minute period for 18-month-old control and *Acsl6^–/–^* males (M) and females (F); *n* = 6–10. (**J**) Total beam breaks in metabolic chambers of 2-month-old control and *Acsl6^–/–^* males (left) and females (right); *n* = 6–13. (**K**) Latency to fall from the rotarod over 6 trials, 3 per day; *n* = 6. Data are shown as the mean ± SEM; *by genotype, ^&^Q1 versus Q2–Q4, *P* ≤ 0.05 by 2-tailed Student’s *t* test.

DHA is predicted to improve memory and prevent dementia. Therefore, memory tests were performed to assess behavioral outcomes in DHA-deficient *Acsl6^–/–^* mice. During Y-maze testing, the *Acsl6^–/–^* mice had significantly fewer spontaneous alternations indicating impaired short-term spatial working memory (Figure 3A). During the Barnes maze (BM) test, no genotype effect was observed for learning index or latency to reach the target (Figure 3, B and C), but *Acsl6^–/–^* mice spent less of their time in the target quadrant 1, indicative of a minor defect in spatial learning and memory (Figure 3D). In the novel object test, no genotype effect was observed for novelty preference or discrimination index, suggestive of intact cognition and recognition memory (Supplemental Figure 1, A and B). Together, these data suggest that ACSL6 deficiency impairs short-term spatial memory.

During the memory tests, *Acsl6^–/–^* mice also exhibited a significant hyperlocomotion phenotype. Specifically, during the BM test, both male and female *Acsl6^–/–^* mice traveled further and froze less (Figure 3, E and F; and Supplemental Figure 1, C and D). During the Y-maze test, *Acsl6^–/–^* mice had more arm entries, indicating hyperactivity (Figure 3G). Increased activity also manifested as increased maximum speed during an open field test (Figure 3H). Open field test did not show indicators of anxiety in *Acsl6^–/–^* mice that traveled to a similar extent as did the controls in the center of the open field (Figure 3I). The activity in metabolic cages showed that both male and female *Acsl6^–/–^* mice had increased ambulatory activity (Figure 3J). During rotarod testing, aged, 18-month-old *Acsl6^–/–^* mice developed a reduced latency to fall (Figure 3K), suggesting reduced motor coordination, a function under cerebellar control. Together, these data support a hyperlocomotion phenotype in *Acsl6^–/–^* mice characterized by increased distance, increased speed, and fewer freezing bouts.

### ACSL6 is a major contributor to cerebellar membrane DHA.

**Figure 4:**
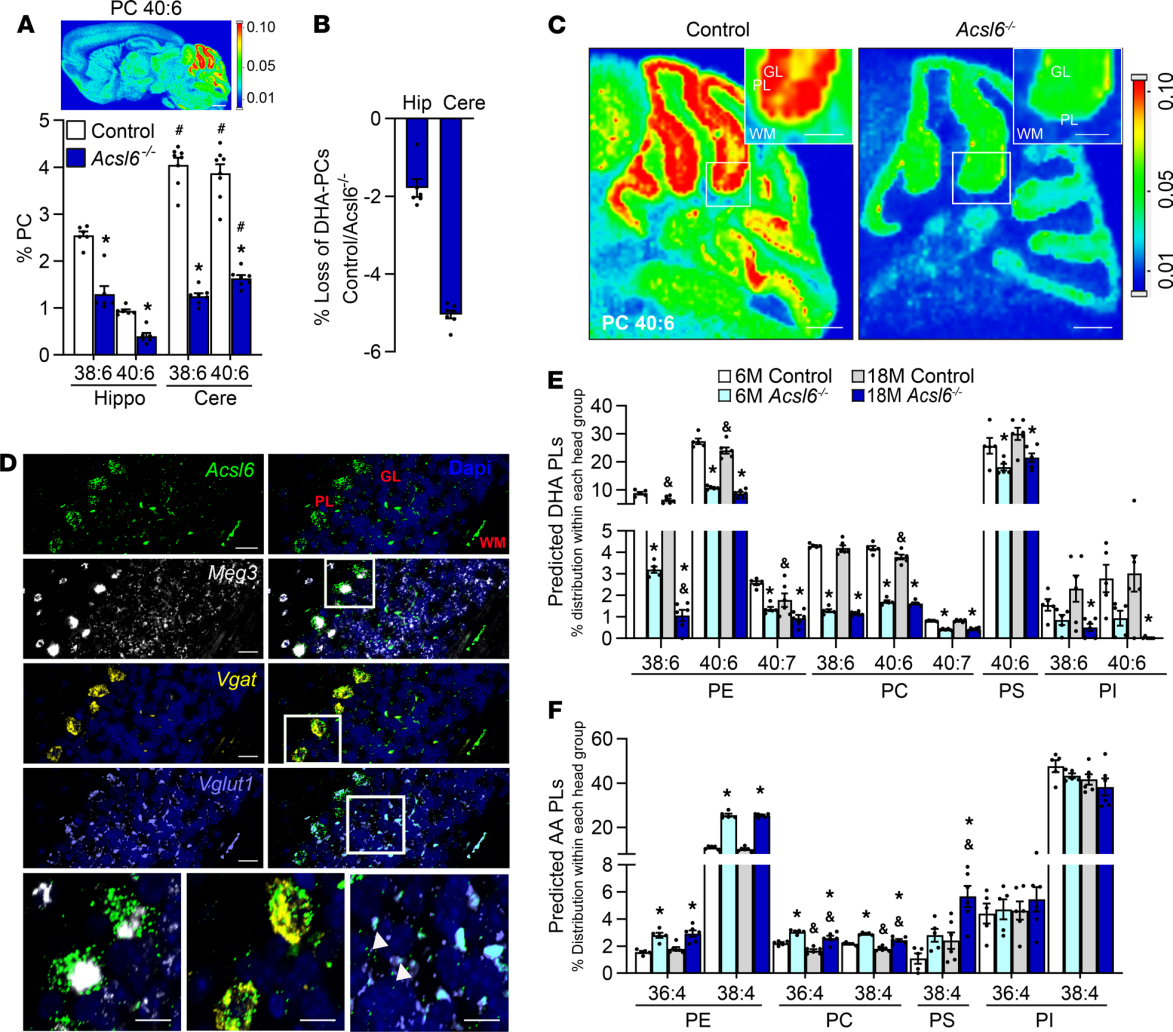
*Acsl6* deletion results in DHA deficiency in the cerebellum during aging. (**A**) Percent of total intensity for DHA-containing PCs (38:6 and 40:6) in hippo and cere, *n* = 6, with MALDI inset of PC 40:6 (*m/z* = 872.5566, [M + K]^+^) in control mice. (**B**) Percent loss of DHA-containing PCs in the *Acsl6^–/–^* hippocampus (Hip) and cerebellum (Cere) relative to control mice. (**C**) Lipid imaging by MALDI of the predicted DHA-containing PC 40:6 (*m/z* = 872.5566, [M + K]^+^) of control and *Acsl6^–/–^* cerebellum (scale bars: 500 μm). GL, granular layer; PL, Purkinje layer. (**D**) *Acsl6* detection by smFISH in cerebellum and inset: blue, nuclei; green, *Acsl6;* white, *Meg3;* purple, *Vglut1;* and yellow, *Vgat*. Arrows indicate *Acsl6*^+^ cells, scale bars: 20 μm; inset, 10 μm. (**E** and **F**) Distribution of phospholipids in 6- or 18-month-old control and *Acsl6^–/–^* cerebellum; *n* = 5–6. Data are shown as the mean ± SEM; *by genotype within age, ^&^by age within genotype, and ^#^compared with hippocampus, *P* ≤ 0.05 (**A**) by 2-tailed Student’s *t* test and (**E** and **F**) by 2-way ANOVA with Sidak’s post hoc test.

Motor activity is controlled in part by the cerebellum, a brain region that we previously showed is highly enriched with ACSL6 (17). Lipid imaging and phospholipid lipidomics demonstrated that DHA-enriched PCs (38:6 and 40:6) were higher in abundance in the cerebellum relative to the hippocampus (Figure 4, A and B). In the *Acsl6^–/–^* cerebellum, MALDI lipid imaging showed largely depleted DHA-enriched PC40:6 (Figure 4C). Spatially, predicted DHA-enriched PCs are most abundant within the Purkinje and granular cell layers of the cerebellar gray matter (Figure 4C). Next, in situ hybridization demonstrated that the regional distribution of PC40:6 was attributed to *Acsl6* expression. Specifically, *Acsl6* transcripts were evident in the granular cell layer and were highly abundant in glutamatergic neurons (*Vglut1*^+^ cells) and within Purkinje neurons of the Purkinje layer (vesicular GABA transporter–positive [*Vgat*^+^] cells) (Figure 4D). The expression of *Acsl6* in Purkinje and granular cells and the loss of DHA-enriched PCs resulting from *Acsl6* deficiency in these cells demonstrate that ACSL6 determines the abundance of membrane DHA in cerebellar neurons.

The effects of age on membrane lipidome in the cerebellum were next assessed by broad untargeted lipidomics. Both young and old *Acsl6^–/–^* mice exhibited reductions in numerous DHA-enriched phospholipids (Figure 4E). As a consequence of aging, PE38:6 was reduced in both control and *Acsl6^–/–^* mice, and PE40:6, PE40:7, and PC40:6 were only reduced in control mice by aging (Figure 4E). Both young and old *Acsl6^–/–^* mice exhibited increased AA-enriched phospholipids (Figure 4F). Aging resulted in reduced PC36:4 and PC38:4 in both genotypes and increased PS38:4 in *Acsl6^–/–^* mice (Figure 4F). Phosphatidylinositol was the only phospholipid refractory to increased AA in the *Acsl6^–/–^* cerebellum (Figure 4F). These data suggest that the cerebellar neurons are highly enriched with ACSL6 and that the cerebellum is highly susceptible to both aging- and *Acsl6*-dependent alterations in the membrane lipidome.

### Transcriptome profiling reveals multiple roles of ACSL6 in the aging cerebellum.

**Figure 5:**
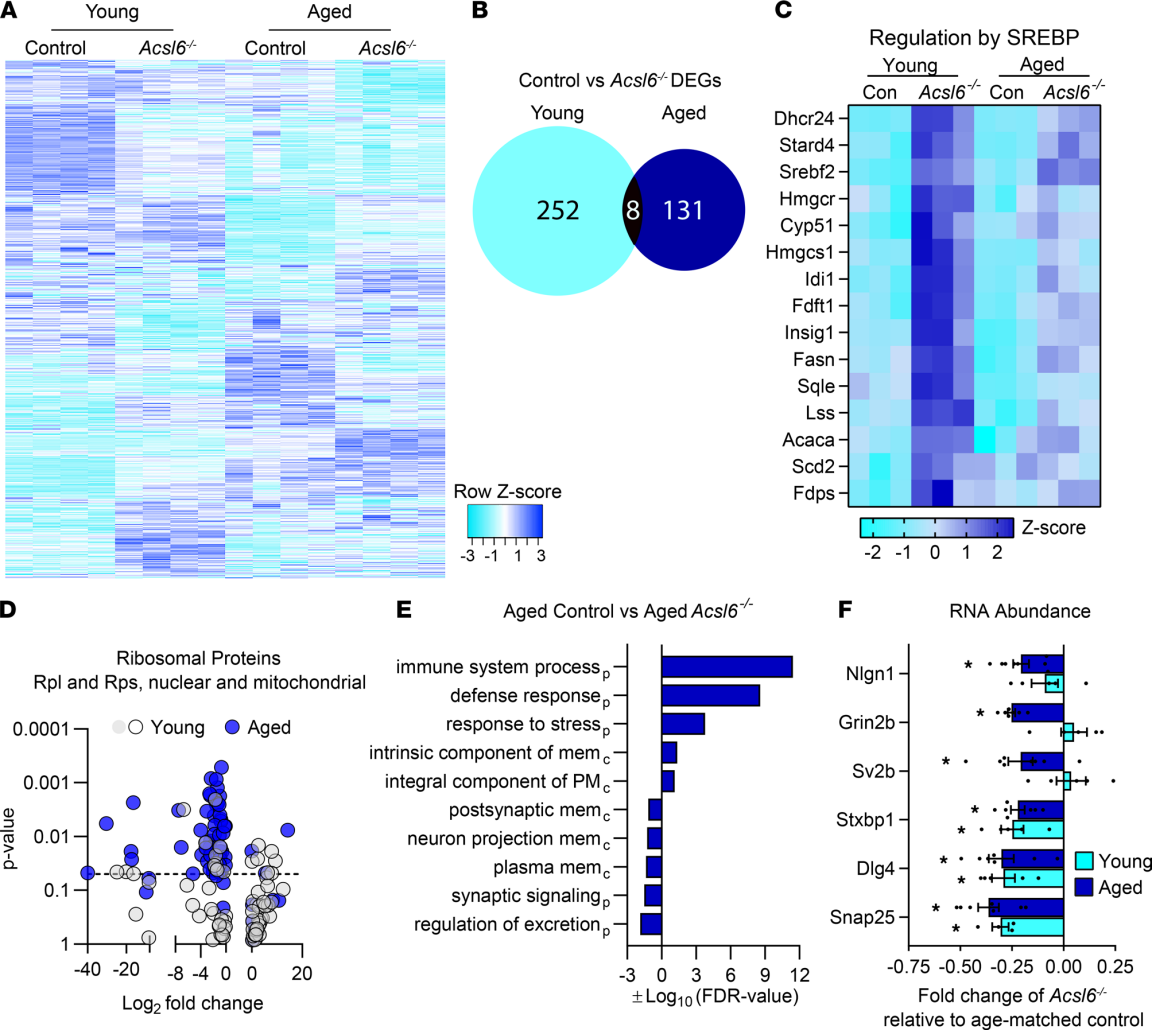
Differential expression analysis in young and aged *Acsl6^–/–^*. RNA-Seq profiling was performed in young (2-month-old) and aged (18-month-old) female control and *Acsl6^–/–^* cerebellum. (**A**) Heatmap of all differentially expressed genes (DEGs) by either genotype within age or age within genotype, *P* ≤ 0.01, graphed according to Pearson’s distance and centroid linkage cluster method. (**B**) Venn diagram of DEGs by *P*adj ≤ 0.05 for genotype comparison in either young or aged mice. (**C**) Heatmap of genes associated with the regulation of cholesterol biosynthesis. (**D**) Volcano plot of aging-effect DEGs of ribosomal protein genes for *Acsl6^–/–^* compared with controls at either 2 (young) or 18 (aged) months of age. (**E**) GO terms from DEGs *P* ≤ 0.05 for *Acsl6^–/–^* compared with controls at 18 months of age (mem, membrane; PM, plasma mem; p, biological process; c, cellular component). (**F**) mRNA abundance of synaptic proteins of young and aged *Acsl6^–/–^* cerebellum, expressed relative to age-matched control mice. Data are shown as the mean ± SEM; *by genotype within age, *P* ≤ 0.05 by 2-tailed Student’s *t* test.

To gain insight into the molecular consequences of *Acsl6* deletion, RNA-Seq was performed using cerebellar tissue from young, 2-month-old and aged, 18-month-old *Acsl6^–/–^* and control mice. Differential genes were identified by comparisons by age within genotype or by genotype within age (Figure 5A). Surprisingly, genes changed (*P* ≤ 0.01) by the loss of Acsl6 in young mice were remarkably distinct from the genotype effect in old mice, with only 2% overlap (Figure 5B), suggesting that the molecular consequences caused by the loss of ACSL6, relative to control mice, are highly susceptible to aging. The mere 7 known genes changed by genotype, independent of aging, included *Acsl6* and genes related to synaptogenesis (*Sparcl1*), endocytosis (*Synj2*), immunity (*C4b*), and cholesterol biosynthesis (*Dhcr24*, *Stard4*, *Srebf2*). Subsequent pathway analysis on a broadened gene set (*P* ≤ 0.05) confirmed the upregulation of the SREBP-regulated cholesterol biosynthesis pathway in both the young and aged *Acsl6^–/–^* cerebellum (Figure 5C and Supplemental Figure 2, A and B). These data agree with the response of SREBP-regulated pathway to changes in membrane DHA content (30–32).

The genes changed by aging in either control mice or *Acsl6^–/–^* mice overlapped by approximately 40% (Supplemental Figure 2). Pathway analysis of the age-affected genes that were similar between the control and *Acsl6^–/–^* cohorts showed increased stress and immune responses and decreased mitochondrial and synaptic genes (Supplemental Figure 2), consistent with the established aging-related phenomenon. However, the pathway analysis of genes changed by age only in the *Acsl6^–/–^* mice revealed additional reductions in genes related to axonal guidance and mitochondria (Supplemental Figure 2). Moreover, numerous ribosomal protein genes were reduced in *Acsl6^–/–^* mice by aging (Figure 5D), a hallmark of natural aging (33, 34). An analysis of genes changed using both Reactome and Gene Ontology (GO) term enrichment analyses revealed that aged *Acsl6^–/–^* mice, when compared with aged controls, had increased responses to stress and of the immune system and reduced membrane-related and synaptic proteins (Figure 5E and Supplemental Figure 2). Synaptic proteins interact with membrane lipids and are therefore susceptible to disruptions in membrane acyl-chain content that alter membrane properties. Reduced gene expression of membrane-related proteins involved in synaptogenesis was confirmed by RT-PCR in 2-month-old *Acsl6^–/–^* cerebellum for *Snap25*, *Dlg4/Psd95*, and *Stxbp1* (Figure 5F). By 18 months of age, these plus additional synaptogenesis-related genes were downregulated, specifically *Sv2b*, *Grin2b*, and *Nlgn1*, all of which have membrane-spanning domains (Figure 5F). While total protein abundance of PSD95 and SNAP25 was not altered in total cerebellar homogenates of the aged *Acsl6^–/–^* cerebellum compared to controls (Supplemental Figure 4), the alterations in transcript levels of these genes suggest dysregulation of synaptic proteins in the *Acsl6^–/–^* cerebellum and agree with previously reported findings linking synaptic proteins to membrane DHA abundance (35, 36). These data suggest that ACSL6 loss differentially impacted gene expression in an age-dependent manner; drove the induction of SREBP; and in response to age, reduced genes related to mitochondrial function, synaptic proteins, axonal guidance, and increased immune response genes.

### Lipid mediator homeostasis is majorly regulated by age and minorly regulated by membrane acyl-chain composition.

**Figure 6:**
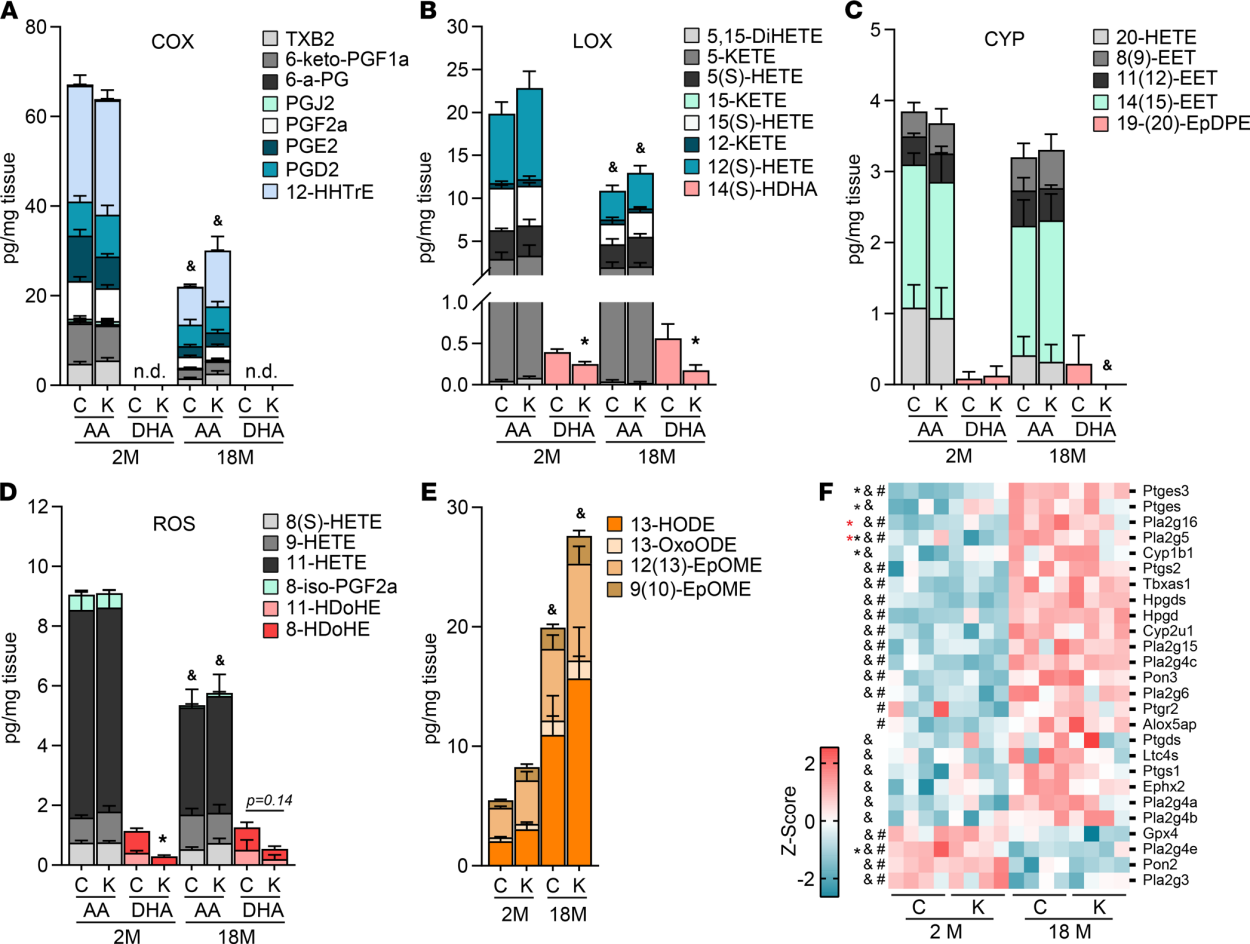
*Acsl6* deficiency minimally impacts fatty acid–derived lipid mediator profile. Stack bar plot of lipid mediators generated from (**A**) cyclooxygenase, (**B**) lipoxygenase, (**C**) cytochrome oxygenase, (**D**) reactive oxygen species, and (**E**) LA-derived lipid mediators in 2- and 18-month-old control (**C**) and *Acsl6^–/–^* (K) cerebellum; *n* = 5–6. The error bars indicate SEM from each lipid mediator. Statistical significance was calculated from the total amount of lipid mediators in the stacked bar, *by genotype within age, and ^&^by age within genotype, *P* ≤ 0.05 by 2-tailed Student’s *t* test. (**F**) Heatmap of significantly DEGs from RNA-Seq (*P* ≤ 0.05) associated with the biosynthesis of lipid mediators plus the phospholipase A2 enzymes in cerebellum; *n* = 4. DEGs were clustered using the Pearson distance correlation measurement method, *(2 M) and *(18 M) by genotype, ^&^(control), and ^#^(*Acsl6^–/–^*) by age, *P* ≤ 0.05 by 2-tailed Student’s *t* test.

Membrane fatty acids are substrates for enzymatic generation of lipid mediators that have multiple downstream regulatory actions including regulation of inflammation. Specifically, lipid mediators derived from enzymatic oxygenation of DHA are generally neuroprotective and proresolution, whereas mediators derived from AA and LA are known to modulate inflammation (37–42). Aging, independent of genotype, decreased AA-derived lipid mediators generated from cyclooxygenases, from lipoxygenases, and from nonenzymatic oxidation by reactive oxygen species (Figure 6, A–D). Conversely, inflammation-promoting LA-derived mediators were increased by age (42), and to a greater degree in *Acsl6^–/–^* compared with controls, suggesting a possible role for LA-derived mediators in age- and *Acsl6^–/–^*-related neuroinflammation (Figure 6E).

Consistent with increased membrane AA in *Acsl6^–/–^* mice, higher levels of AA-derived lipid mediators were observed in aged *Acsl6^–/–^* mice (Figure 6, A–D). The omega-3, DHA-derived lipid mediators were far less abundant than those derived from omega-6 fatty acids. Notably, only 4 of the 14 DHA-derived lipid mediators assessed were detected (Figure 6 and Supplemental Table 2). Despite this low abundance, lipoxygenase and oxidative stress–derived DHA species were significantly lower in *Acsl6^–/–^* mice compared with controls at 2 months of age (Figure 6, B and D). Specifically, the lipoxygenase product 14(S)-HDHA, the precursor of maresin-like specialized proresolvin mediators, was significantly reduced in *Acsl6^–/–^* (Figure 6B). Two nonenzymatic oxidized DHA-derived lipid mediators, 8-HDoHE and 11-HDoHE, were down more than 2-fold in young *Acsl6^–/–^* compared with controls (Figure 6D). Because changes in membrane lipids were not as strongly reflected in the lipid mediator content as expected, we next assessed gene expression of the enzymatic machinery that metabolizes lipid mediators. Surprisingly, we did not observe a genotype effect, but rather we observed age-induced increases in lipid mediator metabolism genes, including several phospholipases that generate substrate, several enzymes that generate lipid mediators, and those that degrade mediators (Figure 6F). While a majority of the genes increased with age, 2 phospholipase A2 genes, paraoxonase 2 and glutathione peroxidase 4, which are associated with aging and oxidative stress (43–45), decreased with age (Figure 6F). Together these data indicate that membrane acyl-chain composition does not greatly impact overall lipid mediator homeostasis or expression of related metabolizing enzymes.

### Loss of ACSL6 promotes neuroinflammation.

**Figure 7:**
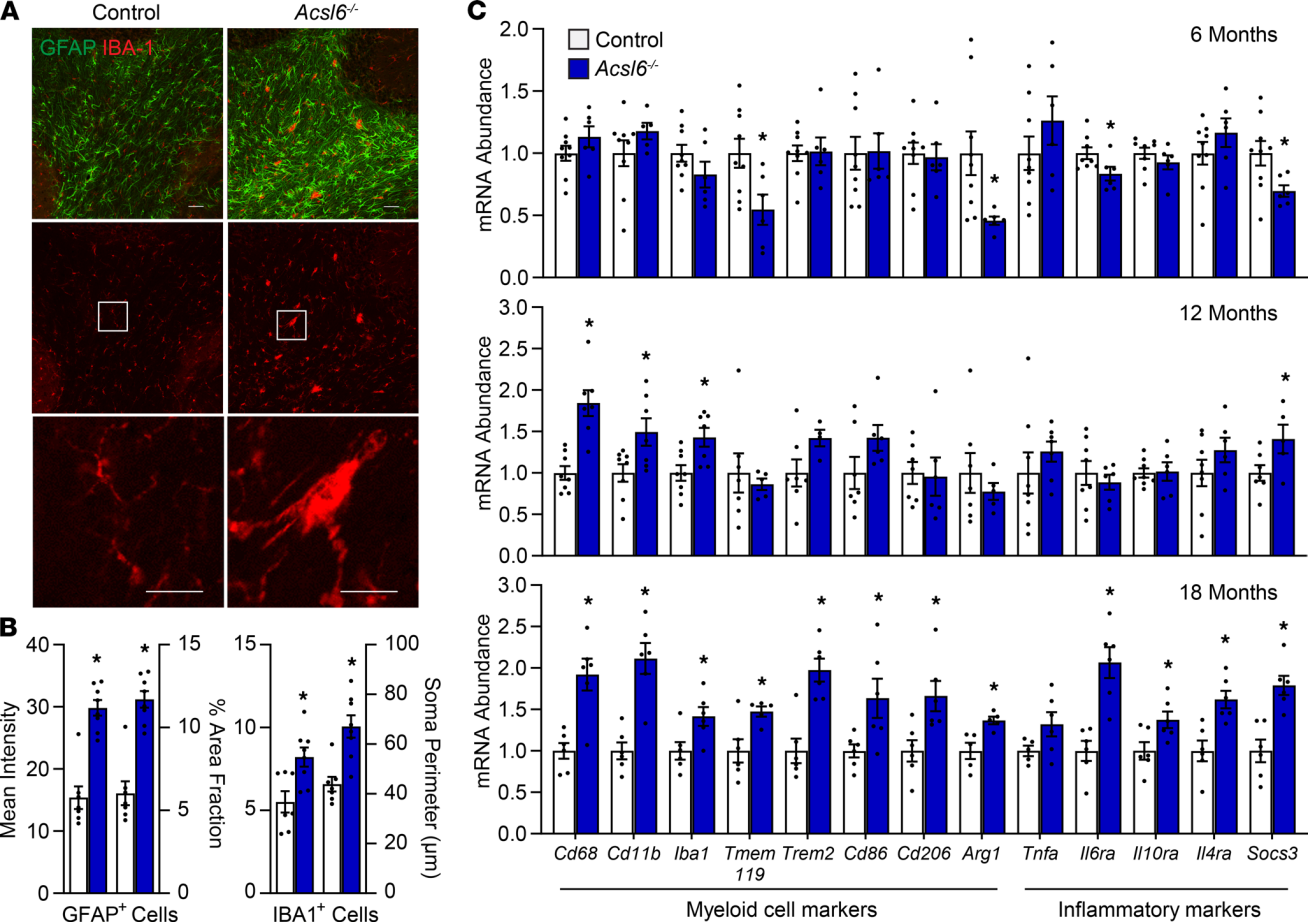
*Acsl6* deficiency results in age-related neuroinflammation. (**A**) Representative immunostaining images of GFAP (green) and IBA1 (red) in 12-month-old male cerebellum (scale bars: 50 μm and 25 μm). (**B**) Mean intensity and percent of area fraction occupied by GFAP^+^ cells in cerebellar white matter and soma perimeter of the 10 largest microglia in cerebellar white matter and percent of area fraction of microglia of 12-month-old control and *Acsl6^–/–^* cerebellum. (**C**) mRNA abundance of inflammatory and myeloid cell markers of 6-, 12-, and 18-month-old *Acsl6^–/–^* male cerebellum normalized to *Rpl22* and relative to control; *n* = 6–8. Data are shown as the mean ± SEM; *by genotype, *P* ≤ 0.05 by 2-tailed Student’s *t* test.

DHA consumption is inversely correlated with age-related diseases afflicting the central nervous system. In agreement, the loss of DHA due to ACSL6 deficiency revealed age-dependent increases in transcriptomic profiles related to immune, defense, and stress responses (Figure 5E and Supplemental Figure 2). To investigate age-related neurology, histological analysis of 1-year-old *Acsl6^–/–^* cerebellum revealed pathological indicators of neuroinflammation. Specifically, aged *Acsl6^–/–^* cerebellum showed enlarged ionized calcium-binding adaptor protein-1–positive (IBA1-positive) and increased GFAP-positive cells (Figure 7A). The area occupied and signal intensity of GFAP^+^ cells in the cerebellar white matter were increased by approximately 2-fold in 1-year-old *Acsl6^–/–^* compared with controls (Figure 7B). For IBA1^+^ cells, the soma perimeter and area occupied were approximately 2-fold greater than controls in the *Acsl6^–/–^* cerebellum (Figure 7B). The increase in neuroinflammatory pathology did not coincide with apparent neurodegeneration as indicated by no change by genotype in aged brain weight, width, or length and by similar cerebellar protein signal for NeuN, the neuronal cell marker, and Calbindin, the Purkinje cell marker (Supplemental Figure 4, E and F). The effect of age on this neuroinflammatory phenotype was assessed by genes that serve as markers for myeloid cells and inflammation across aging. By 12 and 18 months of age, myeloid cell and inflammatory markers were significantly increased in *Acsl6^–/–^* relative to age-matched controls (Figure 7C). These data demonstrate age-responsive microgliosis and/or astrogliosis, as well as increases in pro- and antiinflammatory responses in the *Acsl6^–/–^* cerebellum (Figure 7C).

Unlike *Acsl6^–/–^* mice, and in agreement with minimal impact of astrocytic ACSL6 on cerebellar membrane DHA content, 12-month-old *Acsl6^G–/–^* did not show increased gene expression of myeloid cells or inflammation markers (Supplemental Figure 3, A and B), suggesting that loss of ACSL6 in astrocytes alone does not initiate an age-related inflammatory response. In summary, these data demonstrate that ACSL6-mediated membrane DHA deficiency in cerebellar neurons induces characteristics of aging, including reduced expression of genes related to synaptic activity, membrane components, ribosomal machinery, bioenergetics, and immune/stress responses that culminated in age-related pathological and molecular hallmarks of neuroinflammation and gliosis.

## Discussion

In this report, we show that ACSL6 deficiency in mice presents with age-related neuropathology akin to early-onset aging. The neuropathology and neuroinflammation were readily detected in the cerebellum, a brain region that contains a high abundance of ACSL6 and membrane DHA, suggesting that regions most highly enriched with membrane DHA are impacted the greatest by ACSL6 deficiency. ACSL6 is downregulated in or has identified single nucleotide polymorphisms associated with age-related neurodegenerative diseases (46–48). Thus, the loss of ACSL6 function or availability of its preferred substrate DHA over aging could contribute to age-induced neurological diseases and disorders.

Consistent with an age-related phenomenon, *Acsl6^–/–^* mice transcriptomes were highly influenced by age, and the mice only developed indications of neuroinflammation and pathology with aging. Specifically, the age-dependent increase in reactive glial pathobiology and expression of numerous inflammatory and myeloid markers from 6 to 12 to 18 months of age was evident in the *Acsl6^–/–^* cerebellum. The loss of membrane DHA and the increase in membrane AA had a surprisingly limited impact on steady-state lipid mediator content. It is possible that these minimal effects are due to a lack of proinflammatory challenges to the mice, thereby limiting detection of mediators (49, 50). In relation, we found no evidence by our smFISH or from public databases to suggest that ACSL6 is expressed in microglia, and we showed herein that astrocytic ACSL6 did not regulate DHA metabolism. Thus, ACSL6 deficiency does not cause a DHA deficit in glial cells, arguably the major cell type driving lipid mediator metabolism, thereby potentially limiting the impact of ACSL6 on lipid mediator homeostasis. Because ACSL6 expression is identified as astrocyte enriched in numerous databases (20, 24–28), we were surprised that the loss of ACSL6 in astrocytes had minimal impact on brain membrane lipid composition. However, these databases use cells derived from animals early in development, failing to capture transcripts, such as *Acsl6*, that present in late-stage mature neurons. Our smFISH clearly demonstrates an abundance of *Acsl6* transcripts in neurons. These data agree with the adult human brain RNA-Seq data set from the Allen Brain Atlas showing robust expression of *ACSL6* across a large variety of adult neuron subtypes (51). Importantly, we resolved the mechanistic explanation underlying the difference between astrocytic and complete ACSL6-KO effects on lipid content by demonstrating that astrocytes are enriched with the Y-gate domain but not the DHA-preferring F-gate (29). Thus, we demonstrate that ACSL6 in neurons is DHA preferring whereas ACSL6 in astrocytes is not. Our observation that ACSL6 loss in astrocytes minimally impacts brain lipid content and phenotype suggests that astrocytic ACSL6 does not majorly contribute to brain lipid content and does not elicit the early-onset aging observed in the total-body KO mice.

In relation to cerebellar neurological distress, hyperlocomotion was observed in nearly every test performed on *Acsl6^–/–^* mice across the life span. While the manifestation of hyperlocomotion was not identical (i.e., fewer pauses, faster speed, farther distance traveled) it was consistent, nonetheless. These behavioral abnormalities could be associated with altered neurotransmission due to alterations in membrane properties. Specifically, the kinked nature of DHA contributes to membrane properties such as flexibility, packing defects, and deformation (52, 53). As such, the loss of membrane DHA stands to impair processes such as membrane vesicle cycling at neural synapses for neurotransmission and membrane-protein interactions that are dependent upon the length and hydrophobicity of the membrane bilayer (54–57). As such, a strong match was visualized between ACSL6 expression in neurons by smFISH to that of membrane DHA depletion by MALDI in *Acsl6^–/–^*, suggesting that cerebellar neurons are most susceptible to suffer the consequences of ACSL6 loss. In agreement, GO term analysis was highly enriched with membrane components, coinciding with reductions in synaptoproteome in the cerebellum. Synaptoproteome deregulation has been previously reported as an effect of DHA deficit during aging and is rescued with DHA replenishment, suggesting a direct role for DHA in maintaining adequate synaptic protein levels (35, 36, 58). Here, ACSL6-mediated DHA loss reduced gene expression of synaptic proteins as early as at 2 months of age, suggesting that alterations in synaptic proteins could chronically deregulate synaptic transmission, leading to long-term age-related stress and subsequent neuroinflammation in aged *Acsl6^–/–^* mice.

Motor activity is also largely controlled by dopaminergic control centers, and we previously reported an increase in tyrosine hydroxylase terminal density in the striatum of ACSL6-deficient mice (17). Dopaminergic neuronal projections are particularly abundant in the striatum, and dopamine plays a strong role in regulating motor activity, suggesting a possible link between ACSL6 and dopamine. Our smFISH demonstrated colocalization of *Acsl6* to dopaminergic, tyrosine hydroxylase-positive neurons, and a depletion in membrane PUFAs in this region, visualized by lipid imaging (JME, unpublished observations). Thus, impaired motor function in *Acsl6^–/–^* mice could also be contributed by deficits in dopaminergic membrane PUFAs. Indeed, diseases associated with imbalances in the dopaminergic system, such as Parkinson’s disease, schizophrenia, depression, and attention deficit hyperactivity disorder, have a strong link to DHA. For instance, meta-analysis indicates that DHA supplementation trials have shown relatively consistent and effective benefits for patients with attention deficit hyperactivity disorder (59). Moreover, low levels of DHA and DHA-derived lipid mediators are associated with the pathophysiology of several diseases, including Parkinson’s, multiple sclerosis, and Alzheimer’s (60–62). *ACSL6*-specific mutations have been linked to schizophrenia, and ACSL6 expression is downregulated in patients with Parkinson’s disease and relevant models (46, 47, 63–66). In agreement with a role for DHA in protection against Alzheimer’s disease and dementia, ACSL6-deficient mice had impairments in memory. Since high cholesterol is a strong risk factor for Alzheimer’s disease, whereas DHA is a protector (67, 68), it is possible that these 2 risk factors are linked due to an ACSL6 deficiency–induced DHA deficit impact upon SREBP-mediated increases in cholesterol biosynthesis genes, a finding also reported in other models of DHA deficit (30, 69, 70). Thus, low DHA may increase the risk for Alzheimer’s directly by a gap in DHA’s neuroprotective actions and indirectly due to low DHA’s impact on cholesterol biosynthesis. Together, these data suggest that ACSL6 is involved in providing the adequate lipid environment for maintaining motor-controlling mechanisms of the central nervous system.

In summary, we show that ACSL6 is a major contributor to membrane DHA across the brain throughout the life span. The loss of ACSL6 results in impaired spatial memory, hyperlocomotion, and early-onset neuroinflammation in the aging cerebellum. Additionally, these data suggest that transcriptional control of synaptic proteins is dysregulated in response to altered membrane acyl-chain composition in a manner that precedes neurological impairments. Ultimately, this work demonstrates that *Acsl6* is a key regulator of brain DHA metabolism that confers neuroprotection during aging.

## Methods

### Animals.

Mice were maintained on a 12-hour light/12-hour dark cycle and had ad libitum access to chow (Teklad Global 18% protein rodent diet, Envigo) and water. Chow diet essential fatty acids were not of marine sources and were free of DHA. *Acsl6*^flox/flox^ mice were bred to CMV- or GFAP-Cre transgenic mice (The Jackson Laboratory catalog 006054 and 024098) to generate germline global (*Acsl6^–/–^*) or astrocyte-specific (*Acsl6*^G–/–^**) KO mice, respectively and as previously described (17).

### Behavioral tests.

Locomotor activity of 2- to 3-month-old mice was recorded after 2 days of acclimation in metabolic chambers using an infrared light beam system integrated to an indirect calorimetry system (CaloSys V2.1, TSE Systems) (71). Open field locomotor activity and exploratory behavior of 18-month-old mice were assessed in an open arena (62 × 62 × 46 cm) over a 10-minute period. Activity was recorded and analyzed with the ANY-maze video tracking system (Stoelting Co.). The BM was used to assess hippocampus-dependent spatial learning and memory as previously described (72). The BM consisted of a 1.22-meter-diameter circular platform with 18 holes equally spaced along the outer edges. A dark escape box was placed underneath one of the holes. The BM was conducted over a total of 5 days, including 4 training days, each with 4 trials separated by 15–20 minutes, and 1 testing day. Prior to the first trial each day, mice were acclimated for 30 minutes in the dark. The mouse was placed in the center of a platform inside a dark plastic cup. After 1 minute, the cup was lifted and the mouse was given 3 minutes to find and enter the escape box. If the mouse failed to find the box after 3 minutes, it was guided to it. On day 5, a probe trial was performed in which the escape box was removed and the mouse was allowed to explore for 90 seconds to search for the escape box. Performance was recorded and analyzed with the ANY-maze video tracking system. The learning index was calculated by subtracting the average latency to reach the escape box on day 4 from day 1 (Learning index > 0 means the mouse learned). A novel object recognition test was used to assess recognition memory. Two-month-old mice were placed inside a (25 × 25 cm) box and allowed to explore 2 identical objects (familiar) for 10 minutes. Six hours later, 1 object was replaced with a novel object, and the mice were placed back into the box to explore for 10 minutes. The discrimination index was calculated as follows: (Time in novel – Time in familiar)/(Time in novel – Time in familiar). The Y-maze spontaneous alternation test was performed to assess spatial working memory. Two-month-old mice were placed in a Y-shaped maze composed of 3 equidistant arms and allowed to freely explore for 5 minutes. The alternation percentage was calculated by dividing total alternations by the total number of possible triads (Total alternations/[total entries – 2]) × 100.

### Molecular.

Total tissue RNA isolation was performed using TRIzol (Life Technologies), quantified by NanoDrop 1000 spectrophotometer (Thermo Fisher Scientific), and used to synthesize cDNA with High Capacity cDNA Reverse Transcriptase (Applied Biosystems). Real-Time PCR was performed using SYBR Green Master Mix (Bio-Rad) and primers for the target genes and analyzed using the QuantStudio 3 Real-Time PCR System (Applied Biosystems). Gene expression was normalized to the average Ct values of the housekeeping gene *Rpl22* and expressed as 2^-ΔCT^. For RNA-Seq, cerebellar RNA was isolated using TRIzol and sent for standard RNA-Seq and initial bioinformatics analysis to Genewiz. Libraries were prepared via the Poly(A) selection method and sequenced on 1 lane of the Illumina HiSeq with 2 × 150 bp configuration. Raw sequences were evaluated for quality and trimmed using Trimmomatic v.0.36. Quality trimmed reads were mapped to the *Mus musculus* GRCm38 reference genome available on ENSEMBL to create BAM files using the STAR aligner v.2.5.2b. Unique gene hit counts from the reads that fell within exon regions were calculated using the featureCounts function of the Subread package v.1.5.2. RNA-Seq data have been deposited into the National Center for Biotechnology Information’s Gene Expression Omnibus database under accession number GSE175571. Differential gene expression (DEG) analysis was performed using DESeq2. Comparisons by age (2 versus 18 months) and genotype (control versus *Acsl6^–/–^*) were performed, and the Wald test was used to calculate *P* values and log_2_ fold changes. Thresholds were set for *P* values of less than 0.05. Enriched pathways of DEGs were calculated using REACTOME version 72, and the top significant pathways with a minimum of 10 genes and belonging to the second, third, and fourth hierarchical levels were plotted (73). If pathways from the same category but different hierarchical levels had gene overlap, then the pathway with the lowest hierarchical level containing more than 90% of the genes was selected. Analysis for generating the Venn diagram was performed using Venny 2.1 (74). An unbiased analysis of all the samples was performed to obtain normalized counts and generate the heatmaps. Heatmapper (Wishart Research Group, University of Alberta) was used to cluster genes employing the Pearson distance correlation measurement (75). GO terms were identified using Gene Ontology enRIchment anaLysis and visuaLizAtion tool (GOrilla) (76). For smFISH, HiPlex RNAScope was performed on whole brains from adult mice fixed by immersion in 4% paraformaldehyde (PFA) at 4°C, washed in a graded sucrose series (10%, 20%, 30%), embedded in OCT, frozen, and cryosectioned (5 mm). A series of sections were stained with Nissl to locate the relevant brain slice according to the Allen Mouse Brain Atlas. Twelve-plex smFISH was performed on fixed frozen tissue sections according to manufacturer recommendations using the RNAscope HiPlex8 Reagent Kit and RNAscope HiPlex12 Ancillary Kit (Advanced Cell Diagnostics) to detect transcripts from the following genes: *Acsl6* (584161), *Meg3* (527201), *Slc17a7/Vglut1* (416631), and *Slc32a1/Vgat* (319191). FISH detection was performed using the RNAscope HiPlex Alternative Display Module (catalog 30040) and DAPI counterstain with an AxioImager M1 microscope equipped with 20×/0.8 NA and 63×/1.3 NA objectives and an AxioCam MRm (Carl Zeiss Microscopy). Images from multiple rounds were combined using RNAscope HiPlex Registration Software (catalog 300065). Probes targeting a panel of housekeeping gene mRNAs (RNAscope HiPlex12 Positive Control Probe-Mm; catalog 324321) served as positive controls, and an irrelevant bacterial gene was the negative control (ACD catalog 324341), which were labeled on adjacent sections.

### Lipidomics.

Total fatty acid composition was determined on extracted and fatty acid methyl esters (FAME) prepared using a 1-step extraction/methylation method as described in detail elsewhere (77). Hippocampal samples were treated with an aqueous solution (CH_3_OH/2,2-Dimethoxypropane/H_2_SO_4_ = 8.5:1.1:0.4, v/v/v) and an organic solution (heptane/toluene = 6.3:3.7, v/v) at 80°C for 2 hours. FAMEs were reconstituted in heptane and stored at 20°C until analysis. FAMEs were positively identified by high-resolution capillary gas chromatography covalent adduct chemical ionization tandem mass spectrometry with a GCMS TQ8050 triple-quadrupole mass spectrometer equipped with a prototype solvent-assisted chemical ionization device interfaced to a GC QP2010Plus gas chromatograph (Shimadzu Corp.). FAMEs were quantified using a separate QP2010 GC with a Flame Ionization Detector (Shimadzu Corp.). Response factors were measured daily using an external standard and applied to peak areas to yield calibrated weight percentages (78). Specialized lipid mediators were profiled by HPLC tandem mass spectrometry by the University of Colorado, Aurora, Skaggs School of Pharmacy Mass Spectrometry Facility, as described (79). MALDI lipid imaging of flash-frozen whole brains, sliced 10 μm thick, was performed by the Structural Biology Core of National Institute on Drug Abuse/NIH Intramural Research Program, as described (23), using Thermo Scientific MALDI LTQ-XL-Orbitrap and Xcalibur software (Thermo Fisher Scientific) in positive and negative ion mode with a mass resolution of 60,000 in the mass range of 600–1000 Da. The raster step size of 40 μm was used for both the X and Y directions. 2,5-Dihydroxybenzoic acid matrix (at 40 mg/mL concentration in 70% methanol) was sprayed using TM-sprayer (HTX Technologies). The assignment of lipid species identity was based upon accurate mass with mass error of 2 or fewer parts per million (ppm) in positive ion mode and 3.5 or less ppm in negative ion mode. Broad MRM-based lipid profiling was performed with Purdue University’s Bindley Bioscience Center Metabolite Profiling Facility, as described (17). Briefly, lipids were extracted from tissues using the Bligh and Dyer method (80). The lipid phase was dried, resuspended, and injected through a micro-autosampler (G1377A) into a QQQ6410 triple-quadrupole mass spectrometer operated in the positive ion mode and equipped with a Jet Stream electrospray ionization ion source (both from Agilent Technologies). Data were analyzed by calculating the percent distribution for each ion (ion peak of *m/z* intensity/total ion intensity) and identified based on LipidMaps database.

### Immunoblots and histology.

Mouse brain lysates were collected in lysis buffer (50 mM Tris-HCl, 150 mM NaCl, 1 mM EDTA, and 1% Triton X-100). Proteins were electrophoresed in 10% gels, transferred to nitrocellulose membranes, and blocked with 5% milk in Tris-buffered saline, containing 0.1% Tween 20 (TBS-T) for 1 hour at room temperature. Blots were probed for the primary antibodies ACSL6 (1:1000, MilliporeSigma HPA040470), SNAP25 (1:10,000, Abcam ab5666), PSD95 (1:2000, Invitrogen MA1-045), Calbindin (1:1000, MilliporeSigma C9848), NeuN (1:1000, MilliporeSigma ABN78), and β-tubulin (1:1000, MilliporeSigma T0198) in 3% bovine serum albumin (MilliporeSigma) in TBS-T overnight at 4°C, washed, and probed for anti-mouse (926-68070) or anti-rabbit (926-32211) IRDye-conjugated secondary antibodies (LI-COR) for 1 hour at room temperature. Membranes were scanned with LI-COR Odyssey instrument, and protein was quantified using LI-COR software. Brains were collected and fixed in 4% PFA for 7 days and then incubated in 30% sucrose for 7 days at 4°C. Brain coronal sections were cut at 35 μm thickness using a frozen sliding microtome (Microm HM 450, Thermo Fisher Scientific) and stored in cryoprotectant at −20°C until use. Cerebellums were selected, washed 6 times for 10 minutes with PBS at room temperature, blocked in PBS containing 0.3% Triton X-100 (PBS-T) with 10% normal donkey serum (NDS) for 1 hour at room temperature and with primaries (GFAP, Abcam 53554; Iba1, Wako WEE4506) for 24–48 hours in PBS-T with 1% NDS at 4°C, washed in PBS, and subsequently incubated with secondary antibodies in PBS-T with 1% NDS for 2 hours at room temperature (Alexa Fluor 488 anti-goat, catalog 705-545-147, and Alexa Fluor 647 anti-chicken, catalog 703-606-155; Jackson ImmunoResearch). After washing with PBS at room temperature, 6 times for 10 minutes each, the slides were dehydrated through graded alcohol, cleared, and coverslipped. Images were obtained using an inverted Nikon D-Eclipse C1 confocal microscope. GFAP and IBA-1 staining were analyzed using Fiji imaging software. Briefly, for GFAP, the region of interest was delineated, and the threshold background correction was defined using the same automated threshold algorithm for all images to create binary images. Mean intensity and percent of area fraction (area occupied by GFAP^+^ cells/area of white matter × 100) above the set threshold were calculated. For Iba-1, percent of area fraction of IBA-1^+^ cells and the perimeter of the 10 microglia with the largest body size within the cerebellar white matter were calculated.

### Statistics.

Data are shown as the mean ± SEM for each group, unless otherwise specified. Data were analyzed using Prism 9.0 software (GraphPad). The statistical significance was determined using 2-tailed unpaired Student’s *t* test for comparison of 2 groups, 1-way ANOVA with Tukey’s post hoc test for more than 2 groups, and 2-way ANOVA with Sidak’s post hoc test for multivariate analysis. Statistical significance was set at *P* < 0.05, unless otherwise specified.

### Study approval.

All animal experiments were reviewed and approved by IACUCs at Purdue University, West Lafayette, Indiana, USA (Assurance A3231-01), and East Carolina University, Greenville, North Carolina, USA (Assurance A3469-01).

## Author contributions

RFF, ASP, and JME designed and conducted the research studies; VD, ESW, KAL, SNJ, JBE, BPH, JMG, and JTB acquired the data and provided reagents; and RFF and JME analyzed data and wrote the manuscript.

## Supplementary Material

Supplemental data

## Version 1 06/08/2021

Electronic publication
