# Supplementary material for: Acyl-CoA synthetase 6 is required for brain docosahexaenoic acid retention and neuroprotection during aging
Source: JCI Insight. 2021 Jun 8;6(11):e144351. doi: 10.1172/jci.insight.144351 (PMC8262339; doi:10.1172/jci.insight.144351)
Supplement: Supplemental data [file jciinsight-6-144351-s244.pdf]

Supplemental table and figure legends:

Table S1. Hippocampal fatty acid profile. Percent of total fatty acids (%,w/w) in 6-month or 18-month old control, *Acsl6*$^{G-/-}$, and *Acsl6*$^{-/-}$ hippocampus, n=3-6. The omega-3 fatty acid, eicosapentaenoic acid, was below limits of detection. Data represent mean ± SEM;* compare to control, & within genotype, and # compare to 6-month old *Acsl6*$^{-/-}$, p≤0.05 by Student's t-test.

Table S2. Cerebellar lipid mediator profile. Lipid mediator levels (pg/mg tissue) in 2- and 18-month-old control and *Acsl6*$^{-/-}$ cerebellum, n=5-6. Data represent mean ± SEM;* by genotype, $ by age, p≤0.05 by Student's t-test. Lipoxygenases (LOX); Cytochrome oxygenase (CYP); Epoxy hydrolase (EH); Non-enzymatic autooxidation (NA); Cyclooxygenases (COX); Reactive oxygen species (ROS); Glutathione-S-transferase (GST).

Figure S1. Behavior Tests. (A) % preference for the familiar (F) and novel (N) objects and (B) discrimination index during the novel object recognition test for 2-month old control and *Acsl6*$^{-/-}$ males, n=9. (C) Distance and (D) freezing bouts spent in each quadrant during the probe trial of the Barnes Maze for 18-month old control and *Acsl6*$^{-/-}$ females, n=9-10. Data represent mean ± SEM;* by genotype, $ within genotype, & within genotype and different from quadrant 1, p≤0.05 by Student's t-test.

Figure S2. Differential expression analysis in young and/or aged control and *Acsl6*$^{-/-}$. RNA-seq profiling was performed in 2- (Young) and 18- (Aged) month old female control and *Acsl6*$^{-/-}$ cerebellum, n=4. (A) Venn diagram and REACTOME pathway analysis of significantly DEGs or pathways comparing control to *Acsl6*$^{-/-}$ at either 2 or 18-months of age. (B) Venn diagram and REACTOME pathway analysis of significantly DEGs or pathways comparing young and aged control or *Acsl6*$^{-/-}$ mice.

Figure S3. Effect of astrocytic *Acsl6* loss on lipid content and inflammation. (A) Lipid imaging by MALDI of the predicted DHA-containing PC 40:6 (m/z=872.5566, $[M+K]^+$) of control and *Acsl6*$^{G/-}$ cerebellum (Scale bars: 500μm). (B) mRNA abundance of inflammatory and myeloid cell markers from 12-month old (12M) control and *Acsl6*$^{G/-}$ male cerebellum normalized to housekeeping (average of *Rpl22* and *Tbp*), n=6. Data represent mean ± SEM; * by genotype, p≤0.05 by Student's t-test.

Figure S4. Indicators of neuronal abundance. (A) Representative immunoblot and (B) quantification of SNAP25 and PSD95 normalized to β-tubulin in 18-month old control and *Acsl6*$^{-/-}$ cerebellum, n=8-9. Brain (C) weight, (D) length, and width from 12-month old control and *Acsl6*$^{-/-}$, n=7-8. (E) Representative immunoblot and (F) quantification of NeuN and Calbindin normalized to β-tubulin in 18-month old control and *Acsl6*$^{-/-}$ cerebellum, n=9.

Table S1.

Fatty acid	Common name	6M Control	6M *Acsl6*$^{G-/-}$	6M *Acsl6*$^{-/-}$	18M Control	18M *Acsl6*$^{-/-}$
16:0	PA	22.7 ± 0.23	21.5 ± 1.27	23.1 ± 0.37	21.5 ± 0.59	22.7 ± 0.45
16:1	Palmitoleic acid	0.48 ± 0.02	0.48 ± 0.04	0.57 ± 0.03*	0.55 ± 0.01$^{\&}$	0.56 ± 0.02
18:0	Stearic acid	23.2 ± 0.35	22.3 ± 0.53	22.8 ± 0.15	23.2 ± 0.13	21.7 ± 0.22$^{\&}$*
18:1n9	OA	14.1 ± 0.05	16.8 ± 1.89	15.8 ± 0.64	15.7 ± 0.68	16.5 ± 0.32
18:1n7	Vaccenic acid	3.05 ± 0.14	3.30 ± 0.15	3.32 ± 0.17	3.12 ± 0.10	3.35 ± 0.09
18:2n6	LA	0.64 ± 0.04	1.01 ± 0.04*	1.17 ± 0.09*	0.69 ± 0.04	1.17 ± 0.05*
20:0	Arachidic acid	0.21 ± 0.02	0.34 ± 0.09	0.26 ± 0.02	0.27 ± 0.03	0.26 ± 0.02
20:1	Paullinic acid	0.72 ± 0.15	1.36 ± 0.47	0.87 ± 0.16	1.03 ± 0.20	0.99 ± 0.12
20:2n6	Eicosadienoic acid	0.17 ± 0.01	0.35 ± 0.09*	0.26 ± 0.03*	0.24 ± 0.03	0.25 ± 0.02
20:3n6	DGLA	0.42 ± 0.01	0.47 ± 0.02	0.50 ± 0.03*	0.37 ± 0.02	0.44 ± 0.02*
20:4n6	AA	12.2 ± 0.66	11.8 ± 0.98	13.9 ± 0.70	12.5 ± 0.32	14.0 ± 0.37*
22:0	Behenic acid	0.19 ± 0.03	0.33 ± 0.12	0.24 ± 0.03	0.24 ± 0.03	0.21 ± 0.03
22:1	Erucic acid	0.08 ± 0.01	0.18 ± 0.06	0.12 ± 0.02	0.25 ± 0.06$^{\&}$	0.19 ± 0.02$^{\&}$
22:2n6	Docosadienoic acid	0.18 ± 0.02	0.26 ± 0.06	0.24 ± 0.03	0.25 ± 0.03	0.67 ± 0.49
22:4n6	Adrenic acid	2.55 ± 0.10	2.70 ± 0.17$^{\#}$	3.28 ± 0.07*	2.94 ± 0.12$^{\&}$	3.47 ± 0.10*
24:1	Nervonic acid	0.59 ± 0.11	1.31 ± 0.59	0.98 ± 0.23	1.13 ± 0.21	0.97 ± 0.11
22:5n3	DPA	0.16 ± 0.01	0.16 ± 0.01	0.15 ± 0.01	0.15 ± 0.00	0.14 ± 0.01*
22:6n3	DHA	18.4 ± 0.31	15.4 ± 1.89	12.5 ± 0.36*	15.9 ± 0.60$^{\&}$	12.5 ± 0.24*
SFA		46.2 ± 0.33	44.4 ± 0.55*$^{\#}$	46.4 ± 0.35	45.2 ± 0.63	44.8 ± 0.63
MUFA		19.0 ± 0.85	23.4 ± 3.18	21.6 ± 1.22	21.8 ± 1.20	22.5 ± 0.58
PUFA		34.7 ± 0.79	32.2 ± 2.63	32.0 ± 0.89	33.0 ± 0.70	32.6 ± 0.57
PUFAn-3		18.6 ± 0.31	15.6 ± 1.89	12.6 ± 0.36*	16.0 ± 0.60$^{\&}$	12.6 ± 0.24*
PUFAn-6		16.2 ± 0.77	16.6 ± 0.80	19.4 ± 0.83*	17.0 ± 0.29	20.0 ± 0.48*
n-6:n-3 ratio		0.87 ± 0.05	1.09 ± 0.10$^{\#}$	1.53 ± 0.08*	1.07 ± 0.04$^{\&}$	1.59 ± 0.05*

Table S2.

Compound	Pathway	Precursor	2M Control	2M Acsl6$^{-/-}$	18M Control	18M Acsl6$^{-/-}$
13-HODE	15-LOX	LA	2.07 ± 0.28	3.06 ± 0.42	10.95 ± 3.29[&]	15.69 ± 4.28[&]
13-OxoODE	15-LOX	LA	0.31 ± 0.07	0.45 ± 0.16	1.22 ± 0.36[&]	1.51 ± 0.34[&]
12(13)-EpOME	CYP	LA	2.49 ± 0.13	3.65 ± 0.73	5.98 ± 1.15[&]	8.10 ± 1.43[&]
9(10)-EpOME	CYP	LA	0.62 ± 0.07	1.09 ± 0.25	1.78 ± 0.28[&]	2.32 ± 0.45[&]
12,13-DiHOME	EH	LA	0.55 ± 0.08	0.69 ± 0.12	0.97 ± 0.22	1.24 ± 0.31
9,10-DiHOME	EH	LA	0.28 ± 0.05	0.38 ± 0.08	0.64 ± 0.15[&]	0.86 ± 0.23
9-HODE	NA	LA	2.48 ± 0.28	3.67 ± 0.60	14.19 ± 3.98[&]	19.47 ± 5.13[&]
9-OxoODE	NA	LA	0.45 ± 0.06	0.62 ± 0.21	2.04 ± 0.45[&]	2.10 ± 0.44[&]
EKODE	NA	LA	0.24 ± 0.04	0.41 ± 0.06*	0.58 ± 0.04[&]	0.61 ± 0.10
13-HOTrE	15-LOX	ALA	0.02 ± 0.00	0.02 ± 0.00	0.03 ± 0.01	0.02 ± 0.01
9-HOTrE	NA	ALA	0.01 ± 0.01	0.00 ± 0.00	0.01 ± 0.01	0.03 ± 0.02
9-KOTrE	NA	ALA	-	-	-	-
15-HETrE	15-LOX	DGLA	0.18 ± 0.02	0.20 ± 0.03	0.13 ± 0.01	0.20 ± 0.05
5-HETrE	5-LOX	DGLA	0.01 ± 0.01	0.02 ± 0.01	0.02 ± 0.01	0.03 ± 0.02
8-HETrE	ROS	DGLA	0.03 ± 0.01	0.08 ± 0.04	0.01 ± 0.01	0.06 ± 0.06
12-KETE	12-LOX	AA	0.55 ± 0.19	0.77 ± 0.36	0.48 ± 0.24	0.39 ± 0.15
12S-HETE	12-LOX	AA	8.06 ± 1.35	10.61 ± 1.98	3.35 ± 0.62[&]	4.14 ± 0.81[&]
15-KETE	15-LOX	AA	-	-	-	-
15S-HETE	15-LOX	AA	4.96 ± 0.29	4.62 ± 0.37	2.41 ± 0.37[&]	2.93 ± 0.47[&]
15(R)Lipoxin A4	5-LOX	AA	-	-	-	-
20-hydroxy-LTB4	5-LOX	AA	-	-	-	-
5,15-DiHETE	5-LOX	AA	0.05 ± 0.02	0.09 ± 0.02	0.04 ± 0.02	0.02 ± 0.02[&]
5-KETE	5-LOX	AA	2.89 ± 0.77	3.22 ± 1.22	1.92 ± 0.59	2.05 ± 0.42
5S-HETE	5-LOX	AA	3.34 ± 0.20	3.52 ± 0.70	2.68 ± 0.64	3.44 ± 0.36
6-trans-LTB4	5-LOX	AA	-	-	-	-
8,15-DiHETE	5-LOX	AA	0.04 ± 0.04	0.07 ± 0.07	0.00 ± 0.00	0.00 ± 0.00
Lipoxin A4	5-LOX	AA	-	-	-	-
Lipoxin B4	5-LOX	AA	-	-	-	-
LTB4	5-LOX	AA	-	-	-	-
LTD4_NEG	5-LOX/GST	AA	-	-	-	-
LTE4_NEG	5-LOX/GST	AA	-	-	-	-
11B-PGF2a	COX	AA	-	-	-	-
11-dehydro-tbx3	COX	AA	-	-	-	-
12-HHTrE	COX	AA	25.91 ± 2.31	25.57 ± 2.23	8.42 ± 0.58[&]	12.42 ± 3.15[&]
15-deoxy-Delta12,14-PGJ2	COX	AA	0.15 ± 0.02	0.20 ± 0.04	0.03 ± 0.02	0.04 ± 0.02
6-a-Prostaglandin	COX	AA	0.42 ± 0.05	0.35 ± 0.03	0.10 ± 0.01[&]	0.14 ± 0.02[&]
6-keto-PGF1a	COX	AA	9.00 ± 1.65	7.77 ± 1.51	2.10 ± 0.18[&]	2.77 ± 0.65[&]
Carbocyclic Thromboxane A2	COX	AA	0.06 ± 0.03	0.06 ± 0.02	0.03 ± 0.03	0.00 ± 0.00[&]
dinor-11b-pgf2a	COX	AA	-	-	-	-
dinor-6-keto-pgf2a	COX	AA	-	-	-	-
dinor-8-iso-pgf2a	COX	AA	-	-	-	-
PGB2	COX	AA	-	0.04 ± 0.02	-	-
PGE2	COX	AA	10.13 ± 1.36	7.08 ± 0.58	2.30 ± 0.38[&]	3.00 ± 0.57[&]
PGF2a	COX	AA	8.40 ± 0.96	7.33 ± 0.73	2.45 ± 0.27[&]	3.04 ± 0.34[&]

PGJ2	COX	AA	0.61 ± 0.06	0.67 ± 0.10	0.20 ± 0.03[&]	0.27 ± 0.06[&]
Prostaglandin D2	COX	AA	7.61 ± 1.27	9.29 ± 2.08	4.84 ± 1.13	5.82 ± 1.06
tetranor-PGEM	COX	AA	-	-	-	-
tetranor-PGFM	COX	AA	-	-	-	-
TXB2	COX	AA	4.86 ± 0.46	5.59 ± 0.57	1.59 ± 0.19[&]	2.60 ± 0.60[&]
11(12)-EET	CYP	AA	0.40 ± 0.02	0.40 ± 0.04	0.49 ± 0.08	0.46 ± 0.02
14(15)-EET	CYP	AA	2.02 ± 0.16	1.92 ± 0.41	1.83 ± 0.36	1.99 ± 0.37
20-HETE	CYP	AA	1.08 ± 0.13	0.94 ± 0.19	0.42 ± 0.12[&]	0.32 ± 0.10[&]
5(6)-EpETrE	CYP	AA	0.58 ± 0.39	-	-	-
8(9)-EET	CYP	AA	0.35 ± 0.05	0.43 ± 0.09	0.47 ± 0.09	0.53 ± 0.09
11,12-DiHETrE	EH	AA	0.05 ± 0.01	0.05 ± 0.01	0.07 ± 0.02	0.09 ± 0.03
14,15-DiHETrE	EH	AA	0.08 ± 0.01	0.08 ± 0.01	0.10 ± 0.02	0.12 ± 0.02
5,6-DiHETrE	EH	AA	-	-	-	-
8,9-DiHETrE	EH	AA	0.07 ± 0.05	0.09 ± 0.04	0.04 ± 0.04	0.05 ± 0.05
11-HETE	ROS	AA	6.96 ± 0.64	6.84 ± 0.58	3.59 ± 0.61[&]	3.91 ± 0.72[&]
8-iso-15R-PGF2a	ROS	AA	-	-	-	-
8-iso-PGF2a	ROS	AA	0.51 ± 0.08	0.48 ± 0.11	0.07 ± 0.04[&]	0.10 ± 0.05[&]
8S-HETE	ROS	AA	0.76 ± 0.06	0.76 ± 0.05	0.54 ± 0.07[&]	0.74 ± 0.16
9-HETE	ROS	AA	0.83 ± 0.08	1.03 ± 0.19	1.15 ± 0.20	1.01 ± 0.27
12-HEPE	12-LOX	EPA	-	-	-	-
15-HEPE	15-LOX	EPA	-	-	-	-
18-HEPE	15-LOX	EPA	-	-	-	-
5-HEPE	5-LOX	EPA	-	-	-	-
LXA5	5-LOX	EPA	-	-	-	-
14(15)-EpETE	CYP	EPA	-	-	-	-
17(18)-EpETE	CYP	EPA	-	-	-	-
Resolvin E1	CYP/5-LOX	EPA	-	-	-	-
14,15-DiHETE	EH	EPA	-	-	-	-
17,18-DiHETE	EH	EPA	0.09 ± 0.03	0.12 ± 0.02	0.04 ± 0.03	0.05 ± 0.03
11-HEPE	ROS	EPA	-	-	-	-
8-HEPE	ROS	EPA	-	-	-	-
9-HEPE	ROS	EPA	-	-	-	-
14(S)-HDHA	12-LOX	DHA	0.40 ± 0.04	0.25 ± 0.03*	0.56 ± 0.18	0.18 ± 0.07*
7R Maresin-1	12-LOX	DHA	-	-	-	-
7S Maresin-1	12-LOX	DHA	-	-	-	-
10(S),17(S)-DiHDoHE (Protectin DX)	15-LOX	DHA	-	-	-	-
17(R) Resolvin D1	15-LOX	DHA	-	-	-	-
17(S)-HDHA	15-LOX	DHA	0.27 ± 0.17	-	-	-
Resolvin D1	15-LOX	DHA	-	-	-	-
Resolvin D2	15-LOX	DHA	-	-	-	-
Resolvin D3	15-LOX	DHA	-	-	-	-
Resolvin D5	15-LOX	DHA	-	-	-	-
19(20)-EpDPE	CYP	DHA	0.09 ± 0.04	0.13 ± 0.06	0.29 ± 0.18	0.00 ± 0.00*
19,20-DiHDPA	EH	DHA	-	-	-	-
11-HDoHE	ROS	DHA	0.42 ± 0.07	0.00 ± 0.00*	0.51 ± 0.33	0.20 ± 0.15
8-HDoHE	ROS	DHA	0.73 ± 0.09	0.29 ± 0.04*	0.76 ± 0.16	0.35 ± 0.09*

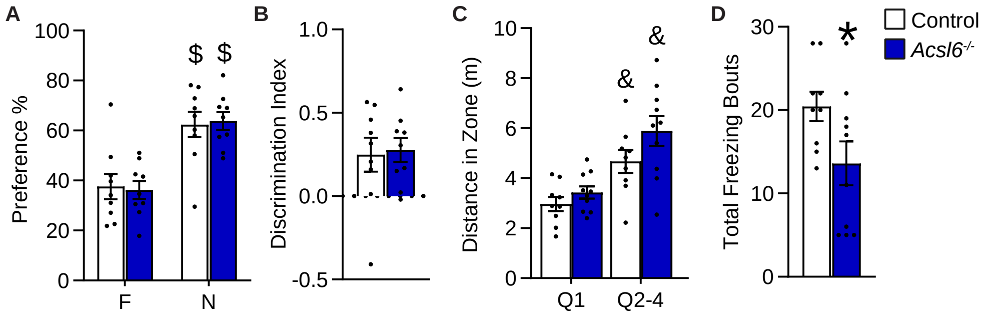
A
B
C
D
Preference %
100
80
60
40
20
0
F
N
$
$
Discrimination Index
1.0
0.5
0.0
-0.5
Distance in Zone (m)
10
8
6
4
2
0
Q1
Q2-4
&
&
Total Freezing Bouts
30
20
10
0
*
Control
Acsl6-/-

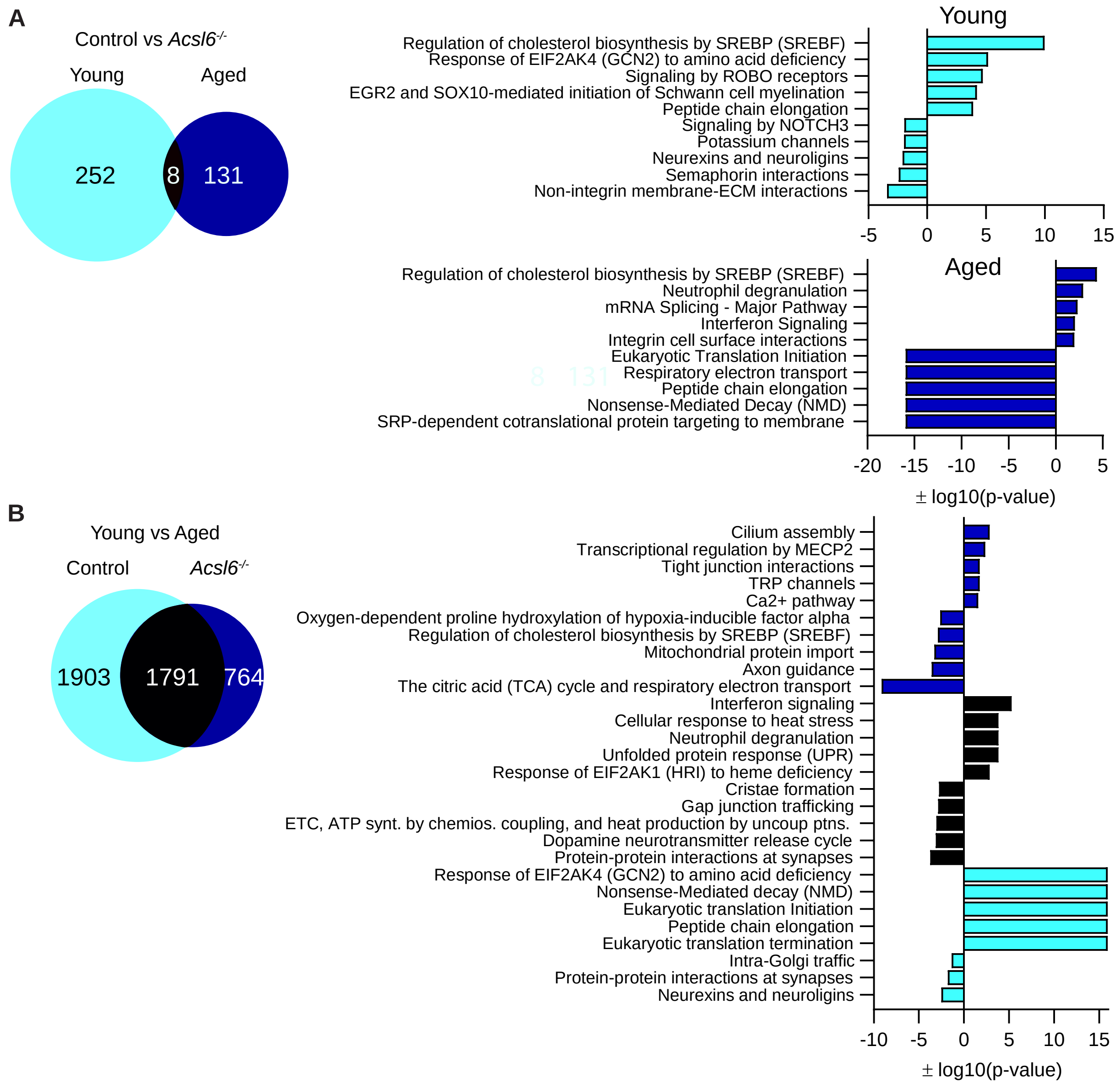

A
Control vs Acsl6-/-
Young
Aged
252
8
131
Young
Regulation of cholesterol biosynthesis by SREBP (SREBF)
Response of EIF2AK4 (GCN2) to amino acid deficiency
Signaling by ROBO receptors
EGR2 and SOX10-mediated initiation of Schwann cell myelination
Peptide chain elongation
Signaling by NOTCH3
Potassium channels
Neurexins and neuroligins
Semaphorin interactions
Non-integrin membrane-ECM interactions
-5 0 5 10 15
Aged
Regulation of cholesterol biosynthesis by SREBP (SREBF)
Neutrophil degranulation
mRNA Splicing - Major Pathway
Interferon Signaling
Integrin cell surface interactions
Eukaryotic Translation Initiation
Respiratory electron transport
Peptide chain elongation
Nonsense-Mediated Decay (NMD)
SRP-dependent cotranslational protein targeting to membrane
-20 -15 -10 -5 0 5
± log10(p-value)
B
Young vs Aged
Control
Acsl6-/-
1903
1791
764
Cilium assembly
Transcriptional regulation by MECP2
Tight junction interactions
TRP channels
Ca2+ pathway
Oxygen-dependent proline hydroxylation of hypoxia-inducible factor alpha
Regulation of cholesterol biosynthesis by SREBP (SREBF)
Mitochondrial protein import
Axon guidance
The citric acid (TCA) cycle and respiratory electron transport
Interferon signaling
Cellular response to heat stress
Neutrophil degranulation
Unfolded protein response (UPR)
Response of EIF2AK1 (HRI) to heme deficiency
Cristae formation
Gap junction trafficking
ETC, ATP synt. by chemios. coupling, and heat production by uncoup ptns.
Dopamine neurotransmitter release cycle
Protein-protein interactions at synapses
Response of EIF2AK4 (GCN2) to amino acid deficiency
Nonsense-Mediated decay (NMD)
Eukaryotic translation Initiation
Peptide chain elongation
Eukaryotic translation termination
Intra-Golgi traffic
Protein-protein interactions at synapses
Neurexins and neuroligins
-10 -5 0 5 10 15
± log10(p-value)

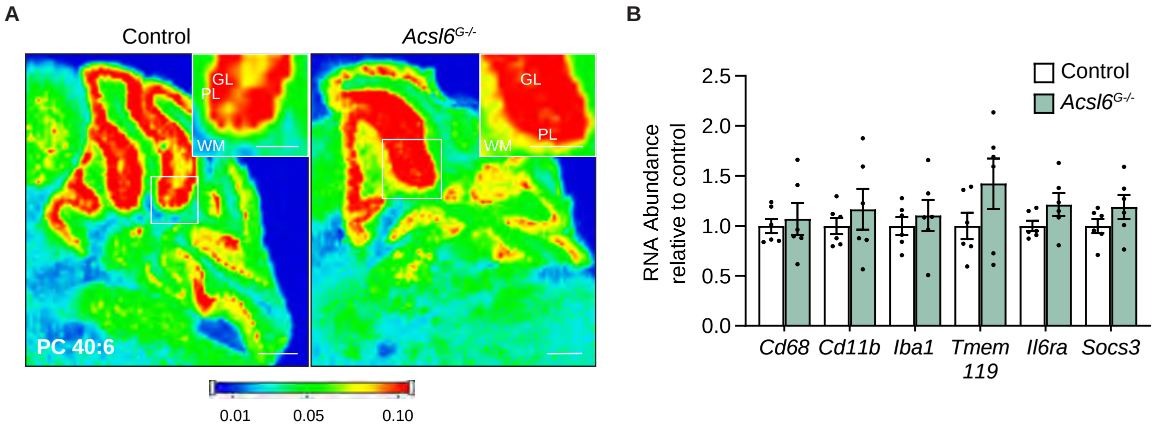

A
Control
Acsl6$^{G-/-}$
GL
PL
WM
PC 40:6
0.01
0.05
0.10
B
RNA Abundance relative to control
2.5
2.0
1.5
1.0
0.5
0.0
Control
Acsl6$^{G-/-}$
Cd68
Cd11b
Iba1
Tmem 119
Il6ra
Socs3

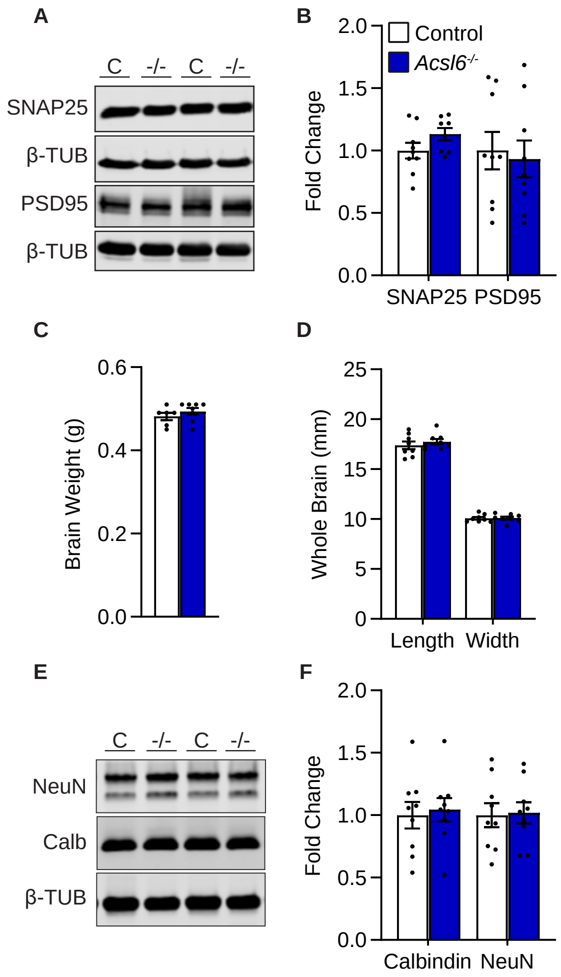

A
B
C
D
E
F
C
-/-
SNAP25
β-TUB
PSD95
NeuN
Calb
Control
Acsl6-/-
Fold Change
Brain Weight (g)
Whole Brain (mm)
Length
Width
Calbindin
2.0
1.5
1.0
0.5
0.0
0.6
0.4
0.2
25
20
15
10
5
0
